# A novel 3D mesenchymal stem cell model of the multiple myeloma bone marrow niche: biologic and clinical applications

**DOI:** 10.18632/oncotarget.12643

**Published:** 2016-10-13

**Authors:** Jana Jakubikova, Danka Cholujova, Teru Hideshima, Paulina Gronesova, Andrea Soltysova, Takeshi Harada, Jungnam Joo, Sun-Young Kong, Raphael E. Szalat, Paul G. Richardson, Nikhil C. Munshi, David M. Dorfman, Kenneth C. Anderson

**Affiliations:** ^1^ Department of Medical Oncology, Jerome Lipper Multiple Myeloma Center, Dana Farber Cancer Institute, Department of Medical Oncology, Boston, MA, USA; ^2^ Department of Medicine, Harvard Medical School, Boston, MA, USA; ^3^ Cancer Research Institute, Biomedical Research Center SAS, Bratislava, Slovak Republic; ^4^ Department of Molecular Biology, Faculty of Natural Sciences, Comenius University, Bratislava, Slovak Republic; ^5^ Biometric Research Branch, Division of Cancer Epidemiology and Prevention, Research Institute & Hospital, National Cancer Center, Goyang-si Gyeonggi-do, South Korea; ^6^ Department of Laboratory Medicine and Translational Epidemiology Branch, Research Institute & Hospital, National Cancer Center, Goyang-si Gyeonggi-do, South Korea; ^7^ Department of Pathology, Brigham and Women's Hospital, Boston, MA, USA

**Keywords:** multiple myeloma, tumor microenvironment, mesenchymal stem cells, 3D model, drug resistance

## Abstract

Specific niches within the tumor bone marrow (BM) microenvironment afford a sanctuary for multiple myeloma (MM) clones due to stromal cell-tumor cell interactions, which confer survival advantage and drug resistance. Defining the sequelae of tumor cell interactions within the MM niches on an individualized basis may provide the rationale for personalized therapies. To mimic the MM niche, we here describe a new 3D co-culture *ex-vivo* model in which primary MM patient BM cells are co-cultured with mesenchymal stem cells (MSC) in a hydrogel 3D system. In the 3D model, MSC with conserved phenotype (CD73+CD90+CD105+) formed compact clusters with active fibrous connections, and retained lineage differentiation capacity. Extracellular matrix molecules, integrins, and niche related molecules including N-cadherin and CXCL12 are expressed in 3D MSC model. Furthermore, activation of osteogenesis (MMP13, SPP1, ADAMTS4, and MGP genes) and osteoblastogenic differentiation was confirmed in 3D MSC model. Co-culture of patient-derived BM mononuclear cells with either autologous or allogeneic MSC in 3D model increased proliferation of MM cells, CXCR4 expression, and SP cells. We carried out immune profiling to show that distribution of immune cell subsets was similar in 3D and 2D MSC model systems. Importantly, resistance to novel agents (IMiDs, bortezomib, carfilzomib) and conventional agents (doxorubicin, dexamethasone, melphalan) was observed in 3D MSC system, reflective of clinical resistance. This 3D MSC model may therefore allow for studies of MM pathogenesis and drug resistance within the BM niche. Importantly, ongoing prospective trials are evaluating its utility to inform personalized targeted and immune therapy in MM.

## INTRODUCTION

Multiple myeloma (MM) is a hematological malignancy characterized by accumulation of malignant plasma cells (PC) in the bone marrow (BM) [1]. In addition to genetic anomalies intrinsic to MM clones, malignant PCs are also influenced by their tumor microenvironment [2]. In particular, fibroblast/stromal-like cells, endothelial cells, immune cells, and the extra-cellular matrix profoundly contribute the behavior of MM cells. Indeed the bidirectional interaction between MM cells and their supporting stromal cells plays a central role in myelomagenesis, mediating resistance to cell death, sustained proliferation, cell homing and invasion, immunosuppression, stemness and self-renewal, as well as angiogenesis, thereby promoting MM progression [3, 4]. Characterizing the efficacy of novel agents therefore should not only assess impact on myeloma cells, but also define effects on the tumor microenvironment.

Specific niches within the tumor microenvironment afford a sanctuary for MM clones due to stromal cell-tumor cell interactions, which confer a survival advantage and drug resistance [4, 5]. Similarities in regulatory signaling molecules and the cell-cell crosstalk interactions between the normal hematopoietic stem cell (HSC) niche and the myeloma niche have been reported [6, 7]. Key common signaling pathways and molecules in both niches include: CXCL12/CXCR4 axis mediating homing and maintenance of HSC/MM cells; IL-6 signaling mediating growth and survival of HSC/MM cells; as well as VCAM1/VLA-4 axis for retention of HSC/MM cells [8–10]. Further defining phenotypic features and molecular signatures of tumor cells in the myeloma niche will provide the framework for development of new treatment strategies targeting the sequelae of MM interactions with the BM milieu.

Several preclinical studies have demonstrated promise of novel anti-MM agents, which has not translated to clinical benefit. This may be due to unfavorable drug pharmacokinetics, but may reflect limitations in drug testing without the tumor microenvironment and/or lack of 3 dimensional (3D) BM architecture [11, 12]. The importance of using *in vitro* 3D, rather than 2-dimentional (2D), models to create an experimental system recapitulating the specialized properties of the MM BM has been demonstrated using: ECM molecules such as collagen and/or fibronectin [13]; specialized scaffolds including gelatin sponges [14] or silk [15]; microfluidic ossified tissues such as osteoblast or plasma [16, 17], as well as RCCS™ bioreactor-based cultures [18]. However, additional studies are needed to closely mimic and investigate the myeloma niche.

Here we introduce a new 3D co-culture *ex-vivo* model to mimic the myeloma niche composed of non-hematopoietic MM-associated mesenchymal stem cells, also known as multipotent stromal cells (MSC), imbedded in a hydrogel 3D system and co-cultured with primary MM patient cells. This model allows for study of cellular components in the myeloma niche including hematopoietic, immune, and tumor cells. In this study, we investigated the role of the tumor microenvironment in the pathogenesis of MM and drug resistance using this 3D co-culture system of MSC with patient BM cells and MM cells. We demonstrated that this 3D co-culture *ex-vivo* system is useful for (i) study of the myeloma biology, especially mechanisms of drug resistance; (ii) evaluation of immune cell subset suppression; (iii) definition of efficacy of anti-MM/experimental drugs; and (iv) evaluation of treatment efficacy against individual patient MM cells to facilitate personalized targeted therapy.

## RESULTS

### Generation of 3D MSC model

**Figure 1:**
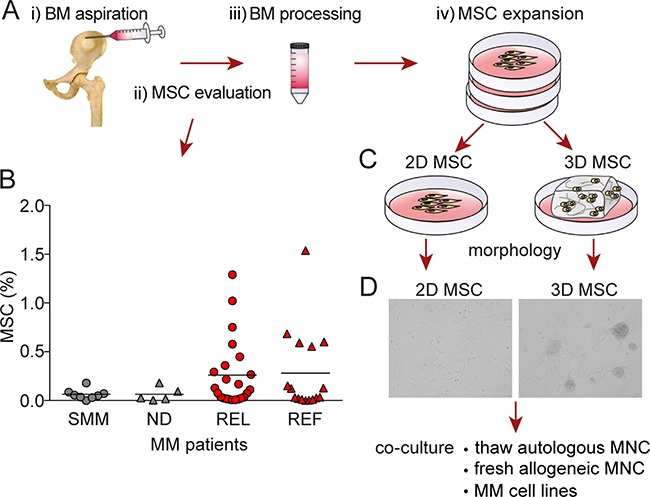
Generation of 3D vs 2D MSC models **A.** Illustration of i) BM aspirate collection, ii) MSC evaluation, iii) BM processing, and iv) MSC expansion. **B.** The distribution of mesenchymal stem cells/multipotent stromal cells (MSC) defined by CD73, CD90 and CD105 profiling was determined in smoldering MM (SMM), newly diagnosed MM (ND), relapsed MM (REL) and relapsed/refractory MM (REF) patient samples by flow cytometry (p=0.563; Kruskal-Wallis one way analysis of variance of ranks). **C.** Generation of conventional monolayer 2D and 3D hydrogel-based MSC models. **D.** A representative picture of MM patient-derived MSC morphology generated in 3D model (right image) compared to monolayer 2D culture (left image) for 5 days. The images were captured with a Leica DFC300Fx camera on an inverted phase contrast Leica microscope using 10X objective and Leica IM50 image-acquisition software Version 4.

To study mesenchymal stem cells/multipotent stromal cells (MSC) at different stages of MM (Figure 1A, ii), we used a multicolor flow cytometry panel to identify cells lacking human lineage markers (CD2, CD3, CD14, CD16, CD19, CD56, CD235a), CD45, CD34 and HLA-DR, but expressing CD73, CD90 and CD105. The percentage of MSCs population was compared in smoldering MM (SMM), newly diagnosed MM (ND), relapsed (REL), and relapsed/refractory (REF) MM (Figure 1B). Interestingly, the MSC population increased in relapsed and relapsed/refractory MM patient samples. To mimic the neoplastic BM microenvironment of MM, we established a novel model of the marrow niche by culturing MM patient-derived MSC *ex-vivo* in a hydrogel-based 3D model (3D MSC) compared with conventional monolayer culture (2D MSC; Figure 1C). In the 3D model, MSC formed compact clusters with active fibrous connections at day 3 to 5 (Figure 1D).

### 3D MSC conserve MSC-specific phenotype and undergo lineage differentiation capacity

**Figure 2:**
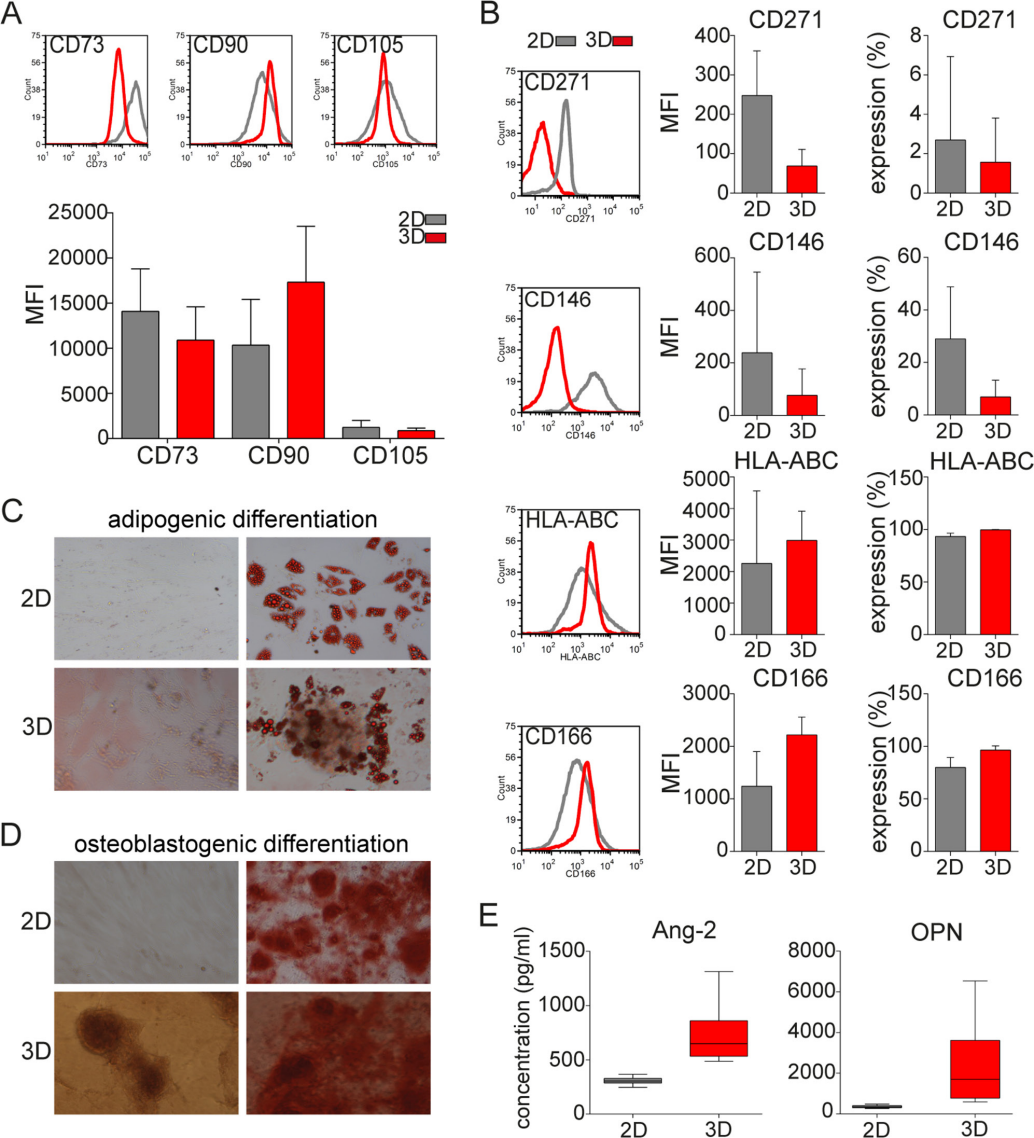
MSC phenotype and lineage differentiation capacity in 3D vs 2D MSC models **A.** Expression (histograms) and mean fluorescence intensity (MFI) of MSC specific markers CD73, CD90, and CD105 on MSC from 52 MM patients after culture for 5 days in 2D or 3D models was assessed by flow cytometry. **B.** Mean fluorescence intensity (MFI) and expression of CD271, CD146, HLA-ABC, and CD166 on MSC in 2D or 3D models was analyzed by flow cytometry. **C.** Representative images of lineage differentiation of MSC in 2D and 3D models are shown. MM patient-derived MSC from 16 MM patients were cultured in either regular media (undifferentiated; left images) or lineage differentiation media (right images). Adipogenic lineage of MSC after 14 days is evidenced by Oil Red O staining of lipid droplets in the cytoplasm (right images). **D.** Osteoblastogenic differentiation of extensive calcium mineralized matrix was assessed after 21 days using 2% Alizarin Red to detect osteoblasts (right images). Images were captured with a Leica DFC300Fx camera on an inverted phase contrast Leica microscope, using 10X objective and Leica IM50 image-acquisition software Version 4. **E.** Production of angiopoietin-2 (Ang-2) and osteopontin (OPN) secreted by MSC in monolayer 2D or hydrogel-based 3D models after 5 days was analyzed by multiplex luminex technology.

MSC are known for the specific phenotype by co-expression of CD73, CD90, and CD105 [19]. MM patient-derived MSC clusters in 3D models highly expressed CD73, CD90, and CD105, as did MSC in 2D model (Figure 2A). Of note, MSC in 3D models revealed significantly decreased expression of CD271 and CD146 than in 2D models (Figure 2B), whereas expression of HLA-ABC and CD166 was similar, only with minimal increases in mean fluorescence intensity in 3D vs 2D models. To assess the differentiation potential of these models, we cultured MSC in 3D versus 2D models with differentiation media towards osteogenic and adipogenic lineages. After culture in adipogenic differentiation media for 14 days, MSC in both 3D and 2D cultures efficiently differentiated into the adipogenic lineage, evidenced by Oil Red staining of lipid droplets in the cytoplasm (Figure 2C). Similarly, MSC grown in 2D versus 3D models with osteoblastogenic media for 21 days formed osteoblasts with extensive calcium mineralized matrix, evidenced by Alizarin red staining (Figure 2D). Moreover, low levels of calcium mineralization of clusters were observed in 3D model, even without osteoblastogenic media. We also assessed production of osteopontin (OPN) and angiopoietin-2 (Ang-2) using multiplex luminex technology. OPN and Ang-2 production was significantly higher in 3D versus 2D MSC models (Figure 2E).

### 3D MSC increase neoplastic BM microenvironment

**Figure 3:**
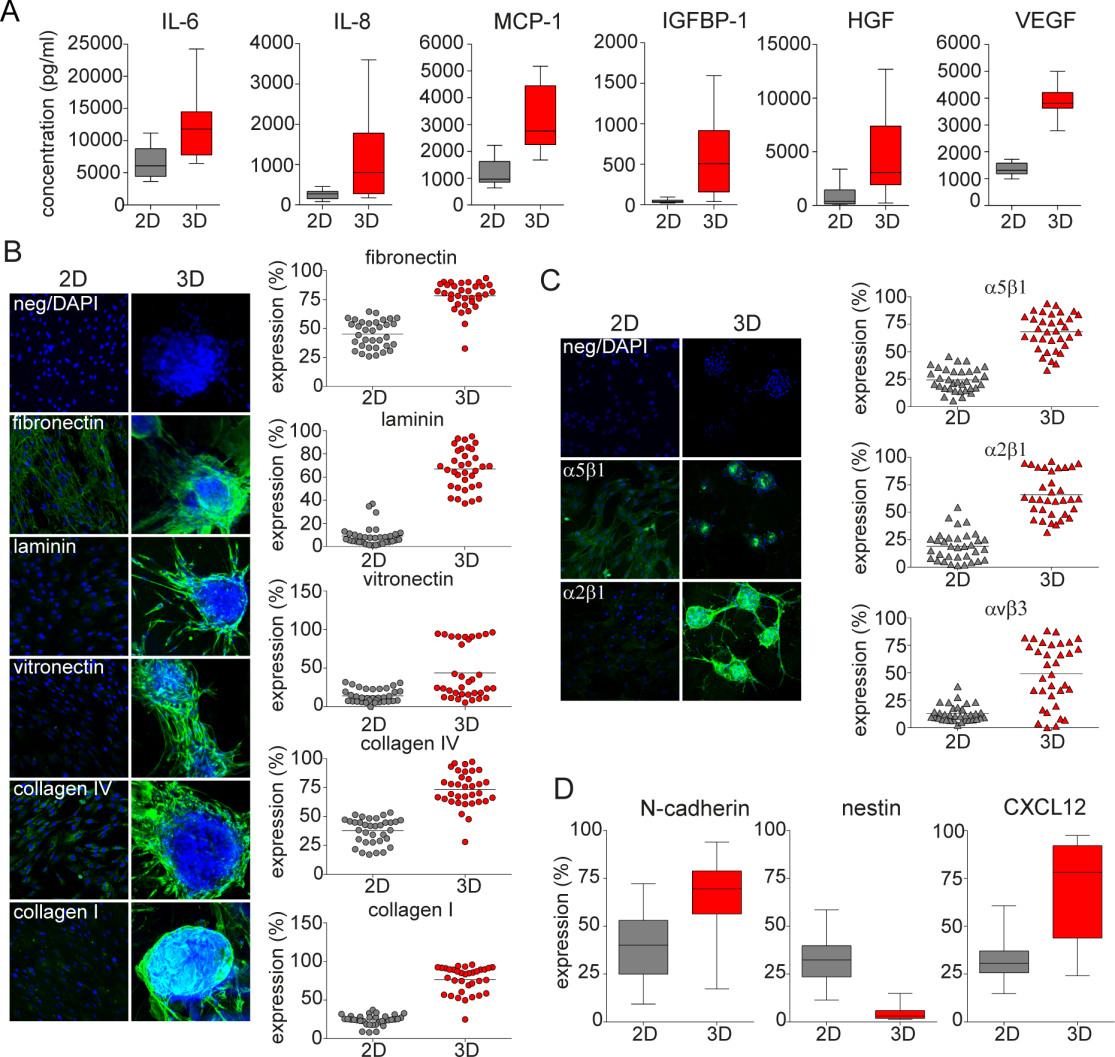
ECM-, integrin- and niche related microenvironment in 3D vs 2D MSC models **A.** Production of cytokines IL-6 (p=0.002) and IL-8, chemokine MCP-1, and growth factors IGFBP-1, HGF and VEGF (p<0.001) secreted by 2D vs 3D MSC conditions after 5 days was determined by luminex technology. **B.** Confocal microscopy analysis (left) and scatter plots from flow cytometry analysis (right) after 5 days of culture of MSC from 34 MM patients show expression of ECM molecules fibronectin, laminin, vitronectin, collagen IV and collagen I in 3D MSC vs 2D models (p<0.001). Green fluorescence represents expression of ECM molecules; nuclei are identified by DAPI staining (blue color). Each dot represents MSCs from one MM patient. **C.** The expression of active integrins α5β1 (VLA-5 receptor for fibronectin, green), α2β1 (VLA-2 receptor for collagens, green) and αvβ3 (VLA-3 receptor for vitronectin) on the surface of 2D vs 3D MSC was determined by confocal microscopy and flow cytometry analyses (p<0.001). Nuclei are identified by DAPI staining (blue color). Each triangle represents MSCs from one MM patient. **D.** Expression of N-cadherin, nestin, and CXCL12 was assessed by flow cytometry analysis on MSC from 34 MM patients after 5 days of culture in 2D vs 3D conditions (p<0.001).

Previous studies suggest that MSCs modulate the MM BM microenvironment by modifying cytokine production [2]. We hypothesized that cytokine secretion by 3D MSC would enhance their ability to support MM cell growth. We measured production of cytokines, chemokines, and growth factors implicated in MM pathogenesis using multiple luminex analysis. Significantly higher production of interleukin 6 (IL-6), interleukin 8 (IL-8), monocyte chemotactic and activating factor (MCP-1), insulin-like growth factor binding protein 1 (IGFBP-1), hepatocyte growth factor (HGF), and vascular endothelial growth factor (VEGF) was observed in 3D versus 2D MSC models (Figure 3A).

### 3D MSC express ECM- and integrin-rich microenvironment

Because the stem cell niche is enriched with extracellular matrix (ECM) molecules promoting expansion of the stem cell pool, we next studied ECM expression in these models. Significantly higher expression of ECM molecules including fibronectin, laminin, vitronectin, collagen IV, and collagen I was evident in 3D versus 2D MSC models, as determined by confocal microscopy analysis (Figure 3B, left). Similarly, in 5 day cultures of MM patient-derived MSC, we observed statistically significantly higher levels of ECM molecules in 3D versus 2D models, as determined by flow cytometry analysis (Figure 3B, right). Consistent with the expression of various ECM molecules, expression of β1 and α4 subunits of VLA-4 integrin were also higher in 3D compared to 2D MSC cultures, as determined by immunofluorescence staining of confocal microscopy and confirmed by flow cytometry analysis (Supplementary Figure S1). Furthermore, formation of dimeric active integrins including α5β1 (VLA-5), α2β1 (VLA-2), and αvβ3 (VLA-3) on the MSC surface when engaged by these ECM molecules, which induces MM proliferation and survival, was also increased in the 3D versus 2D MSC model, as confirmed by confocal microscopy and flow cytometry analysis (Figure 3C).

### 3D MSC form functional myeloma niche

To mimic the functional myeloma niche, key hematopoietic and/or neoplastic niche molecules including N-cadherin and nestin, as well as the interactions of MM cells with MSCs in the niche via CXCL12/CXCR4 axis, were next investigated. MM patient-derived MSC cultured in 3D versus 2D models showed significantly increased expression of N-cadherin and CXCL12, as detected by flow cytometry analysis. In contrast, expression of nestin was significantly decreased in 3D versus 2D MSC models (Figure 3D). Moreover, levels of both soluble Tie-2 receptor (sTie-2) and stem cell factor (SCF), functionally implicated in the HSC niche, were higher in 3D compared to 2D MSC cultures. Similarly, higher levels of human epidermal growth factor receptor 2 (sHER2neu) and soluble epidermal growth factor receptor (sEGFR) were evident after 5 days in 3D versus 2D MSC cultures, assessed using multiplex luminex based technology (Supplementary Figure S2).

### 3D MSC up-regulate bone-formatting genes/form osteogenesis

**Figure 4:**
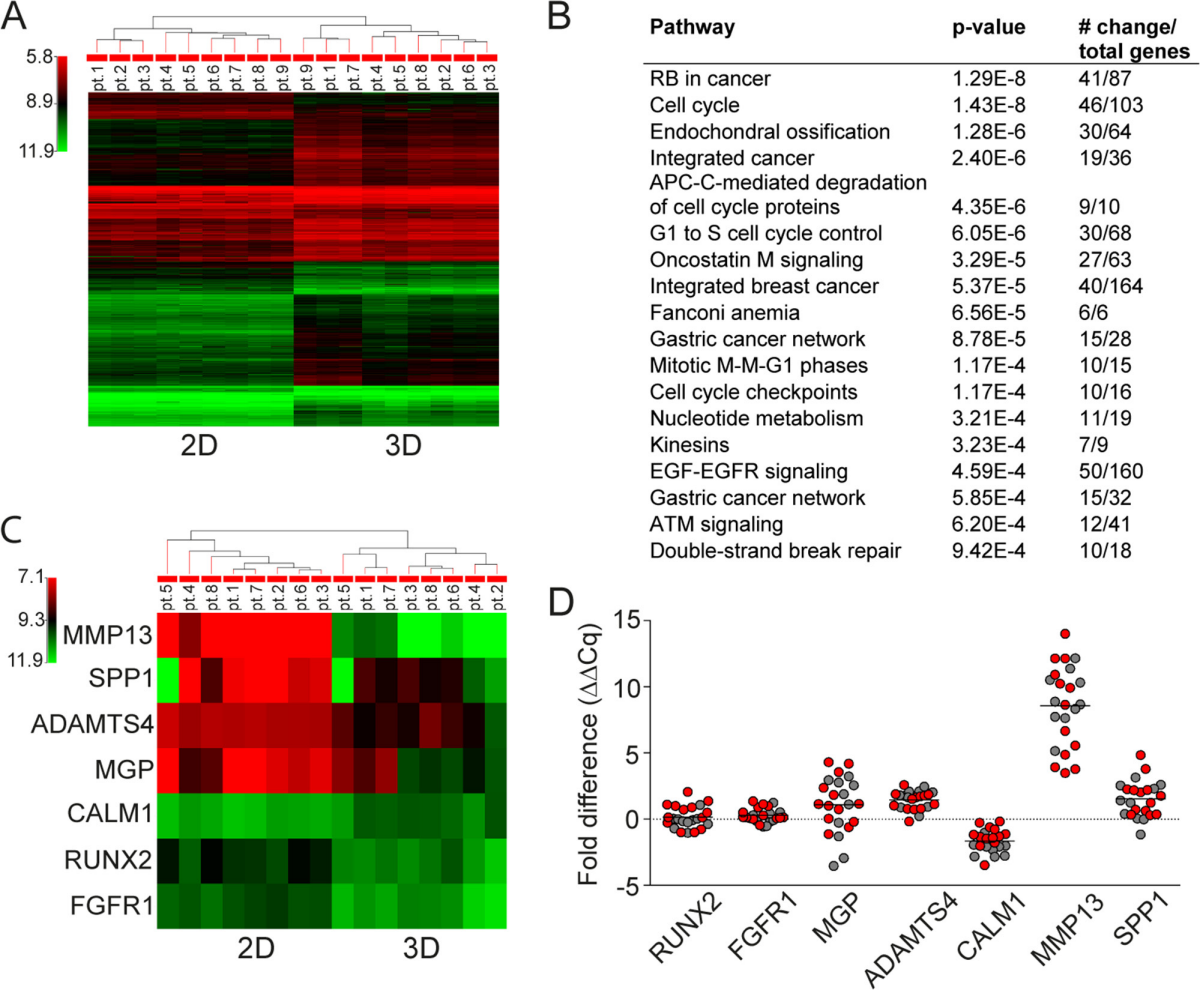
Gene expression analysis in 3D vs 2D MSC models **A.** Unsupervised gene expression analysis using GeneSpring software of MSC obtained from SMM (N=5) and ND (N=4) MM patients after 5 days of culture in 3D vs 2D models. **B.** Major signaling pathways with most significantly modulated genes in 2D versus 3D MSC are listed from highest to lowest p values. **C.** Unsupervised gene expression analysis of the 7 genes (MMP13, SPP1, ADAMTS4, MGP, CALM1, RUNX2, and FGFR1) altered in MSC in 3D vs 2D models obtained from SMM (N=4) and ND (N=4) MM revealed increased endochondral ossification signaling pathway using GeneSpring software. **D.** Fold expression difference of RUNX2 (p=0.425), FGFR1 (p=0.003), MGP (p=0.016), ADAMTS4 (p≤0.001), CALM1 (p≤0.001), MMP13 (p≤0.001), and SPP1 (p≤0.001) genes in 3D vs 2D cultures of MSC from SMM and ND MM (red color, N=13) and REL and REF MM (grey color, N=10) patients were analyzed by real-time RT-PCR analyses using Bio-Rad Software. Normalized fold expression difference was calculated by the ΔΔCq method.

We next compared differences in gene expression analysis in 3D versus 2D MSC models. Unsupervised analyses of gene expression profiles of SMM (N=5) and ND MM (N=4) showed significant differences in 3D versus 2D models, evidenced by two major distinct clusters in heat map (Figure 4A). Several major signaling pathways implicated in cancer, cell cycle- regulation, and adhesion were upregulated in 2D compared to 3D MSC models (Figure 4B). The most significantly upregulated genes in 3D versus 2D MSC were in the signaling pathway mediating endochondral ossification (Figure 4C). The highest upregulation was detected in matrix metallopeptidase 13 (MMP13), followed by bone sialoprotein (SPP1), matrix gla protein (MGP), and ADAM metallopeptidase with thrombospondin type 1motif 4 (ADAMTS4), as well as slightly increased expression of Runt-related transcription factor 2 (RUNX2) and fibroblast growth factor receptor 1 (FGFR1) genes; in contrast, calmodulin 1 (CALM1) was significantly down-regulated. Furthermore, real-time RT-PCR analyses comparing MM patient (SMM, ND, REL, REF; N=23)-derived MSC cultured in 3D versus 2D models confirmed significantly upregulated MMP13, SPP1, ADAMTS4, and MGP with a slight increase of RUNX2 and FGFR1 expression; in contrast, expression of CALM1 was significantly decreased (Figure 4D), consistent with activation of osteogenesis in the 3D MSC model.

### 3D MSC co-culture with BM mononuclear cells/MM cells

**Figure 5:**
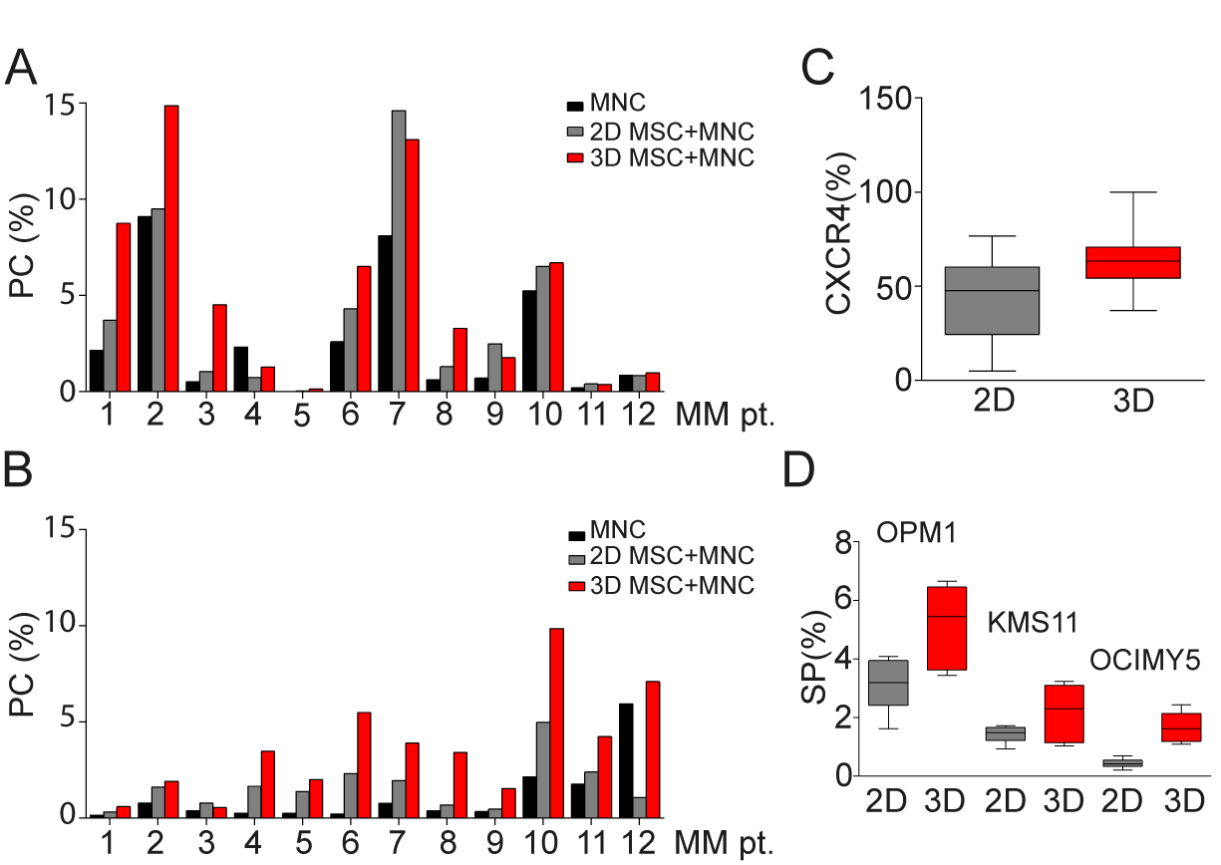
BM primary MM cells co-culture in 3D vs 2D MSC models **A.** Thawed mononuclear cells (MNC) from 12 MM patients were cultured for 7 days together with autologous MSC maintained in culture for 5 days before in 2D vs 3D co-culture conditions. Plasma cells (PC), identified by co-expression of CD38 and CD138 differed before co-culture (black column) and after 7 days in 2D vs 3D co-culture systems (grey and red columns) (p=0.004). Holm-Sidak method revealed significant differences in 3D vs before co-culture (p=0.003). **B.** Freshly isolated allogeneic MNC from 12 MM patients were cultured for 7 days with patient-derived MSC in 2D vs 3D co-culture models (grey and red columns). PC significantly differed, before co-culture (black column) and after 7 days in 2D vs 3D co-culture models (p<0.001), Holm-Sidak method revealed significant differences in 3D vs before co-culture and 3D vs 2D MSC models (p<0.001). **C.** Expression of CXCR4 on both autologous and allogeneic PC (N=24) in 2D vs 3D co-culture models (p=0.013) was evaluated by flow cytometry. **D.** SP cells of MM cell lines, identified by Hoechst 33342 staining of flow cytometry, were measured after 3 days of co-culture of MSC from 12 MM patients in 3D vs 2D conditions with OPM1 (p=0.014), KMS11 (p=0.044) and OCIMY5 cells (p<0.001) labeled with CFSE dye.

**Figure 6:**
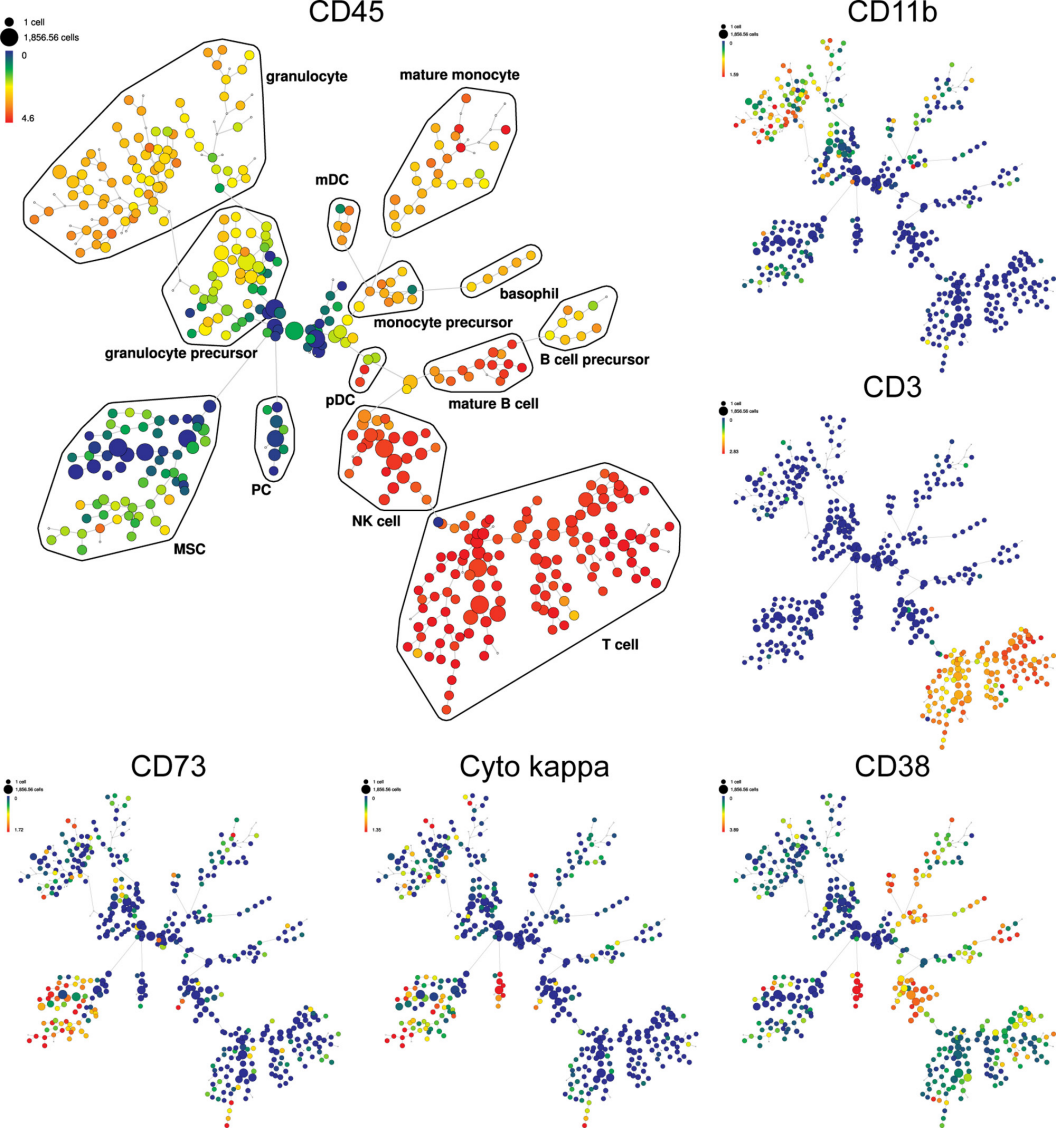
Detection of immune cell subsets in 3D model by SPADE analysis SPADE plots of bone marrow MNC of representative MM patient co-culture with allogeneic MSC in 3D model. SPADE clustering was performed on all samples (N=5) before, and after co-culture in 3D vs 2D models simultaneously to generate a single tree structure for all samples. MNC before co-culture were used as baseline for both 2D and 3D co-culture models. All cell events from each sample were then mapped to the common tree structure. SPADE organized cells to the cluster in the tree by a hierarchy of related phenotype. The size of each node is correlated to the fraction of cells mapping to the node. Each node of the SPADE tree is colored to median expression of the indicated markers: from low (blue color) to high (red color) expression. Unsupervised clustering analyses define the main immunophenotypic populations in SPADE tree, based on selected clustering cell surface markers. Immunophenotypic subpopulation identification was performed manually by grouping of nodes on the basis of the median marker expression level of each node, and based on analysis of the relevant biaxial plots into: T cell (CD3+CD4+CD8+CD7+), NK (natural killer) cell (CD3-CD7+CD45-RA+CD56+/−CD16+/−), B cell precursor (CD38+CD19-CD123+/−), mature B cell (CD38+CD19+), PC (CD45- CD38++CD138+cyto kappa+/cyto lambda-), monocyte precursor (CD33+/−CD11b+/−), mature monocyte (HLA-DR+CD33+CD11b+CD11c+CD15-CD14+), basophil (HLA-DR-CD38+CD123+), mDC (myeloid dendritic cell; HLA-DR+ CD123-), pDC (plasmacytoid dendritic cell; HLA-DR+ CD123+), granulocyte precursor (HLA-DR- CD11b_dim_CD11c_dim_), mature granulocyte (HLA-DR-CD33+/−CD11b+CD11c+CD15+CD16+), and MSC (CD73+CD90+CD105+CD63+). Representative SPADE plots for specific markers are shown, such as main hematopoietic marker CD45, then CD11b, CD3, CD38, cyto kappa and CD73. + = positive

We next co-cultured BM mononuclear cells (MNC) from MM patients (N=12) with MM patient-derived MSC for 7 days in 2D versus 3D models. MNC were either thawed (autologous model; Figure 5A) or freshly isolated (allogeneic model; Figure 5B), evaluated for plasma cells (PC) expression before co-culture (black column), and then seeded either into hydrogel based 3D (red) or monolayer 2D (grey) models in which MSC had been maintained for 5 days in culture. In autologous MNC co-cultures with MSC, we noted increased proliferation of PC in 7 of 12 MM patients in both 3D and 2D models, with slightly higher proliferation rate in the 3D MSC model (Figure 5A). Moreover, MSC in 3D versus 2D conditions co-culture with allogeneic MNC from MM patients (N=12) similarly showed significant increase of PC in 3D versus 2D models in 9 of 12 MM patients (Figure 5B). Further phenotypic characterization of these cells showed that CXCR4 expression was significantly increased on PC co-cultured in 3D versus 2D MSC models (Figure 5C). Furthermore, expression of side population (SP) cells representing “stem-like” features of MM cells [20] was determined by Hoechst 33342 staining and gating on CFSE positive MM cells in co-cultures with MM patient-derived MSC; low Hoechst stained cells were identified as SP cells (Figure 5D). The SP fraction of MM cell lines (OPM1, KMS11 and OCIMY5) was significantly increased in 3D versus 2D MCS co-culture models at 3 days. We also co-cultured MSC in 3D versus 2D models with allogeneic MNC from MM patients (N=5) to assess immune subpopulation distribution using mass cytometry and spanning-tree progression analysis of density-normalized events (SPADE) (Figure 6). No significant differences in immune cell frequencies were detected between 2D versus 3D models; however, decreased T cells and mature granulocytes were detected in 3D versus 2D models (Supplementary Figure S3A). Of note, median expression of immunophenotypic markers on T cells, NK cells (Supplementary Figure S3B); mature B cells, PC (Supplementary Figure S3C); monocyte and their precursors, mDC, granulocytes as well as their precursor (Supplementary Figure S3D); and MSC (Supplementary Figure S3E) significantly differs in 3D versus 2D models. Further evaluation of T cells including T_H_ (helper) and T_C_ (cytotoxic) cell populations as well as both naïve and memory T cells by SPADE analyses revealed that no significant differences in cell frequencies were detected in these models (Supplementary Figure S4A); however, changes in median expression of T cell specific markers were observed between 2D versus 3D models (Supplementary Figure S4B). The similar distribution pattern of immune cell subsets in both models suggests that they may be useful to assess immune profile before and after novel targeted and immune therapies.

### 3D co-culture system confirms MM resistance

**Figure 7:**
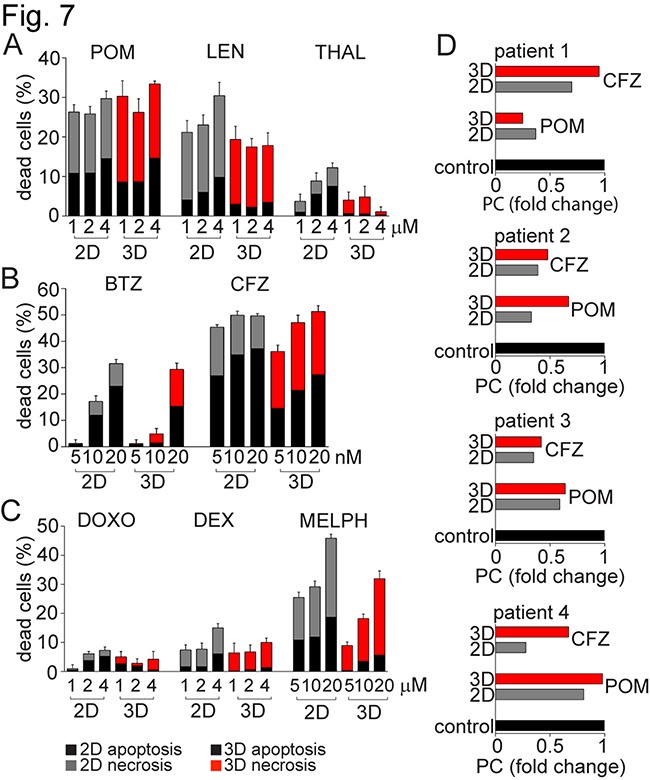
MM resistance to anti-MM agents in 3D vs 2D MSC models MSC from 10 MM patients were maintained in culture for 5 days either in 2D vs 3D culture conditions, and then co-cultured together with plasma cells (PC) labeled with CFSE. These PCs, obtained from the pleural effusion of a MM patient with resistant disease, were treated with **A.** immunomodulatory drugs: pomalidomide (POM), lenalidomide (LEN) and thalidomide (THAL) at concentrations 1, 2 and 4 μM; **B.** proteasome inhibitors: bortezomib (BTZ) and carfilzomib (CFZ) at concentrations 5, 10 and 20 nM; or **C.** conventional anti-MM therapies: doxorubicin (DOXO; 1, 2 and 4 μM), dexamethasone (DEX; 1, 2 and 4 μM), and melphalan (MELPH) at concentrations 5, 10 and 20 μM. After 3 days, cells were stained with AnnexinV/7-AAD and analyzed by a FACS Canto II flow cytometer using a 96-well format. The percentage of apoptotic (AnnexinV+7-AAD-) and necrotic (AnnexinV+7-AAD+) CFSE positive PCs is shown in the graphs. **D.** Freshly isolated MNC from 4 MM patients were cultured with allogeneic MM patient-derived MSC in 2D vs 3D co-culture models, and then treated with POM (4 μM) and CFZ (20 nM) for 7 days. BM samples tested were obtained from patients with MM: patient 1 had relapsed MM (REL) previously treated with CFZ-LEN-DEX; patient 2 had smoldering MM (SMM) who had not been treated; patient 3 had newly diagnosed MM (ND) treated with induction regimen cyclophosphamide-BTZ-DEX; and patient 4 had relapsed/refractory MM (REF), previously treated with POM-BTZ-DEX regimen. Expression of PC was evaluated by flow cytometry, and fold change of PCs relative to control under respective 2D vs 3D co-culture conditions is shown.

To test drug efficacy in the 3D model, MSC from 10 MM patients were cultured in 3D versus 2D models for 5 days, and then allogeneic drug resistant CFSE fluorescent dye-labelled patient PC were added with novel and conventional anti-MM drugs for 3 days. PC co-cultured with 3D versus 2D MSC did not reveal any significant differences after pomalidomide (POM) treatment; however, PC in 3D co-cultures were significantly more resistant to lenalidomide (LEN) and thalidomide (THAL) than in 2D MSC model (Figure 7A). Similarly, resistance to proteasome inhibitor bortezomib (BTZ) was shown in 3D versus 2D MSC model. In contrast to bortezomib, treatment with carfilzomib (CFZ) showed high percentage of apoptotic and necrotic PC in both 2D and 3D co-culture systems (Figure 7B). Resistance to doxorubicin (DOXO) and dexamethasone (DEX) was also observed in both co-culture systems, and resistance to melphalan (MELPH) treatment was observed in the 3D vs 2D model systems (Figure 7C). Similarly, freshly isolated MNC from BM of MM patients (N=4) were cultured with allogeneic MSC in 3D versus 2D conditions, followed by treatment with POM or CFZ for 7 days (Figure 7D). Resistance to POM or CFZ was observed in 3D versus 2D models in MM patients 2 or 4 and 1, respectively. While these data reflect clinical resistance to these agents, ongoing prospective trials are validating their predictive value to conventional, novel and immune therapies in larger patient numbers.

## DISCUSSION

In the BM, specialized microdomains termed “niches” regulate hematopoietic stem cells (HSC) function [21]. These specific HSC niches play essential roles in controlling the balance in hematopoiesis through heterologous cell-cell interactions, secreted factors, extracellular matrix molecules (ECM), physical parameters, as well as immunological and metabolic control. Increasing evidence suggests that, like stem cells, tumor cells do not depend solely on cell-intrinsic events, but instead rely heavily on the microenvironment or “niche” to maintain their growth, survival, and drug resistance [22]. In MM, interactions between malignant plasma cells and stromal/mesenchymal stem cells (MSCs) embedded in an extracellular matrix make up the unique neoplastic BM milieu that promotes the disease development and progression. Therefore, the tumor microenvironment is recognized both as an essential factor in MM biology and target for novel therapeutic strategies [23].

Several novel experimental drugs have not been translated to the clinic due to preclinical drug evaluation involving only tumor cells without consideration of the role of the tumor microenvironment. Therefore, several groups have developed stromal cell-MM cell co-culture models as tools for novel drug screening [11, 12]. However, these models lacked the 3D architecture of the BM, and therefore fail to mimic the *in vivo* niche. More recent studies have elucidated the importance of using 3D, rather than 2D, models to create an experimental system recapitulating the specialized properties of the marrow microenvironment [24]. Specifically, 3D cultures using ECM molecules [13], hyaluronic acid [25], and specialized scaffolds like gelatin sponge [14] or silk [15] have been introduced *in vitro*; however, these models lack mineralization and do not mimic the strength, rigidity, or complexity of *in vivo*. Other more sophisticated/advanced approaches, such as microfluidic ossified tissue [16, 17] or RCCS™ bioreactor-based culture [18], remain very technically challenging. Moreover, several murine humanized MSC-MM cell interaction models have been developed using the SCID-hu model [26, 27], or based on scaffolds such as 3D poly-ε-caprolactone polymeric scaffolds [28] or biphasic calcium phosphate [29]; however, these models are costly, time-consuming, and exhibit drug distribution limitations. Therefore, more studies are needed to develop models of the architecture of BM, facilitating studies of MM pathophysiology and novel drug development.

Several similarities between the normal HSC niche and the MM niche have been described. To develop an experimental system recapitulating the specialized properties of the neoplastic BM microenvironment, we therefore first expanded and modified the HSC niche model [30]. Specifically, we first introduced new 3D co-culture *ex-vivo* models composed of MM patient-derived MSC in hydrogel 3D system for co-culture with patient MM BM cells. In the field of tissue engineering, creating hydrogel based 3D culture systems, a network of interacting polymer chains that are highly hydrated with elasticity similar to natural tissues, with MSCs can achieve tissue-like structures *in vitro*. Importantly, MM patient-derived MSC, which represent the main cellular component of the marrow niche, in the hydrogel based system promote a high degree of mineralization and differentiation and create MM niche-like structures *in vitro.* These patient-derived MSC supply the appropriate niche scaffold for MM growth, survival, and homing. Moreover, MSCs are clonogenic and can be expanded *ex vivo* without any apparent modification in phenotype and loss of function [31] and therefore represent a promising strategy for tissue engineering, repair of damaged tissue, and gene therapy.

Previous reports show that myeloma-associated MSC demonstrate significant abnormalities compared to healthy donors MSCs, including ECM, integrin and cytokine modulation changes [32–34]. It has been reported that there are 0.01-0.001% of MSCs in the healthy bone marrow. Our data showed varied distribution of MSC population (defined by co-expression of CD73, CD90 and CD105 and negative for all hematopoietic markers, such as lineage markers as well as CD34 progenitors) in differently treated MM patients (REL and REF MM patients) compared to untreated MM patient samples in premalignant (SMM) or newly diagnosed (ND) active MM stages. Interestingly, higher MSC population was revealed in treated REL and REF MM samples (0.01-1.3%), suggesting an impact of anti-MM therapies on the tumor microenvironment. Moreover, significantly less (more than 15 times) amount of MSCs (≤1.3%) was observed compared to plasma cells (≤20%, defined by co-expression of CD38 and CD138; data not shown), confirming the necessity for the MSC expansion to study MM disease in their presence.

Three criteria to define MSCs have been determined: adherence to plastic; expression of CD73, CD90 and CD105; and differentiation into three-lineage stages [19]. BM-derived stromal progenitors also have been termed mesenchymal stem cells due to their specific capability of generating multiple mesenchymal lineages [31]. Our data showed that 3D MSC maintained their multi-lineage differentiation potential toward adipocytes and osteoblasts, as well as conserved phenotype defined by co-expression of CD73, CD90 and CD105. Moreover, 3D MSC showed calcium mineralization of clusters supporting differentiation towards the osteoblastogenic lineage, which was functionally confirmed by increased production of OPN and angiopoietin. OPN and angiopoietins are regulators of osteoblast-mediated HSC/MM cells maintenance, and are expressed by osteoblasts [35]. Furthermore, our 3D MSC model showed increased expression of N-cadherin as well as up-regulation of bone-related genes including MMP13, supporting early osteoblast differentiation. Similarly, activation of osteogenesis in the 3D MSC model versus 2D monolayer cultures is associated with significant overexpression of endochondral ossification pathway genes including SPP1, MGP and ADAMTS4. These results are consistent with the observation that pre-osteoblast/early osteoblast precursors increase proliferation of MM cells, in contrast to mature osteoblasts which form mineralized nodules and suppress the growth and proliferation of MM cells [36–39]. Moreover, MSCs are negative for hematopoietic markers CD45, CD34 and other lineage differentiation markers [19]. We here confirmed that the 3D MSC clusters lacked expression of CD45, CD34 and HLA-DR, as well as lineage differentiation markers similar to 2D MSC (data not shown). Furthermore, they display common expression profile markers including CD29, CD49c, CD166 and HLA-ABC, which are similarly detected in both our systems. Nevertheless, significant differences have been shown in expression of CD36, CD106, CD146 and others [40]. Similarly, 3D MSC lacked expression of CD146, which suggests similarities with CD45-CD146-negative expression of MSC located in the endothelial niche [41]. Moreover, CD271 downregulated expression in 3D MSC suggests MSC differentiation from CD271+ progenitors [42]. Therefore, our 3D MSC model mimics the BM niche, allowing for further studies of MM biology and novel therapeutic strategies.

Indirect communication between tumor cells and MSCs is mediated by secretion of growth factors, chemokines, and cytokines. IL-6 remains the prototypical growth and survival factor in MM [43]. In addition to IL-6, other cytokines and/or chemokines also contribute to the cross-talk between stroma and MM cells including IL-8, RANTES (regulated and normal T cell expressed and secreted), IL-1β, TNF-α, IGF-1 (insulin-like growth factor-1) and others [6]. VEGF also modulates growth, survival and migration of myeloma cells in an autocrine manner via VEGFR-1 [44]. Moreover, the tumor microenvironment releases growth factors for myeloma cells including HGF, matrix metalloproteinases (MMPs), fibroblast growth factor-2, MCP-1 (monocyte chemotactic protein-1), and others [45]. Our data showed that MSC in the 3D model support production of pro-inflammatory interleukins IL-6 and IL-8; MCP-1; IGFBP-1; as well as pro-angiogenic growth factors VEGF and HGF involved in progression of MM. Moreover, production of sHER2/neu and sEGFR, growth factors of aggressive cancers, was also assessed in 3D MSC. Our study suggests that recruitment of MM cells and other normal hematopoietic cells and non-hematopoietic cells and their progeny to the MM niche leads to a complex milieu of growth factors, chemokines, and ECM that can form a functional niche. Therefore, deeper understanding the phenotypic features and molecular signatures of the neoplastic BM niche in MM may provide the framework for future development of new treatment strategies targeting biology of both MM cells and their BM niche.

Key signaling regulatory molecules and cell-cell crosstalk interactions involved in MM are directly adopted from the normal HSC niche. The most abundant acellular component of the BM niche is composed of ECMs including fibronectin, laminin, collagen, and hyaluronan. Abnormal ECM or ECM stiffness deregulates the tumor microenvironment by facilitating angiogenesis and inflammation [46]. Moreover, ECMs mediate adhesion, migration, and cell adhesion-mediated drug resistance of MM cells by binding to adhesion molecules [47]. HSC/MM cell adhesion is mediated via the integrin family including very late antigen 4 (VLA-4, composed of α4 and β1 subunits) and VLA-5 (active dimer of α5 and β1 subunits) [48, 49]. Our 3D model showed upregulation of ECM molecules and integrins (α2β1, α5β1, and αvβ3) which are mediators of MM progression and resistance to apoptosis. In addition, the CXCL12-CXCR4 axis signaling pathway plays an important role in MM maintenance and homing, with high expression of CXCL12 on stromal cells acting as a chemoattractant for both HSC/MM cells expressing high level of CXCR4 [50]. In our 3D model, we observed significantly higher expression of CXCL12 on myeloma derived-MSCs and upregulated CXCR-4 expression on plasma cells, as well as significant production of sTie-2 receptor and SCF, reflecting the MM niche.

Normal PC are terminally differentiated and unable to proliferate [51]. Similarly, primary malignant PCs have a low proliferation index *in vitro*, whereas the tumor microenvironment promote MM survival and proliferation [3]. Although MM cell lines can proliferate *in vitro*, they are mostly derived from plasma cell leukemia and have lost BM dependency. Therefore, it is questionable how far we can force the malignant PC to proliferate. Our data showed that both *ex vivo* conventional 2D monolayer and 3D MSC co-culture systems increased proliferation of primary malignant PC, with a higher proliferation index in the 3D model. Moreover, the impact of autologous and allogeneic conditions did not reveal any significant differences in proliferation of PC, suggesting that autologous MM derived-MSC may not be required in the co-culture system. Interestingly, SP fraction representing stem cell-like cells was upregulated in MM cells when co-cultured with 3D MSC versus the 2D system. Finally, CXCR4 was significantly increased on PC in the 3D system and previous data showed CXCR4 expression increased in SP cells in co-culture with MSC [52], suggesting MM homing and adhesion mediating their survival.

Progression of MM is associated with immune suppression that interferes with tumor elimination and an effective antitumor response. Recent evidence indicates that interactions among tumor cells, stromal cells, and immune cells regulate anti-tumor immunity. Specifically, stromal signatures can predict for survival [53], as can immune T cells and macrophages in the tumor microenvironment [54]. Moreover, interactions among the malignant clone, stroma, and immune cell subsets engender an immunosuppressive tumor microenvironment [55]. Here we therefore incorporated not only MSC/stromal and tumor cells in our 3D model, but also immune cells. Applying the CyTOF technology resulted in high-dimensional data that allows for the quantification of phenotypically and functionally distinct immune cell subsets. Importantly, we used an antibody panel tailored to innate and adaptive immune cells, manual gating strategies, as well as clustering algorithm by SPADE approach, to define immune cell subsets. Our results demonstrated that distribution of immune cells can be readily assessed in our 3D MSC co-culture model, which will allow for defining both the role of immune accessory cells and the effects of novel chemo-/immune-based therapies.

Although commonly sensitive to novel therapies, MM cells develop resistance underlying disease relapse. The emergence of resistance may be due to very complex microenvironmental-tumor cell interactions in bone marrow [56]. Therefore, we next evaluated sensitivity of MM cells to IMIDs (THAL, LEN and POM); proteasome inhibitors (BTZ and CFZ); and conventional drugs (DEX, DOXO and MELPH) using our 3D MSC co-culture system. Our results showed significant resistance of MM cells to THAL and LEN; BTZ; and conventional anti-MM (DEX, DOXO and MELPH) therapies reflecting clinical resistance. We next evaluated sensitivity of MM cells from 4 patients to POM or CFZ in this system, and similarly demonstrated profiles reflecting clinical sensitivity versus resistance. These preliminary studies need to be validated in prospective trials, but do suggest that our 3D co-culture system may predict for clinical utility of novel therapies.

In conclusion, in this study we investigated the role of the marrow microenvironment in MM. Interactions among malignant PC, immune accessory cells, and MSC/stromal cells with ECM, integrins, and other niche molecules which mediate disease development and progression were characterized using a hydrogel based 3D *ex vivo* co-culture system. Importantly, the 3D co-culture closely mimics MM marrow physiology and therefore provides a model to now explore growth of MM clones associated with drug resistance and minimal residual disease. Importantly, it may identify novel mechanisms to target to interrupt these processes. Ultimately the 3D co-culture model may assess tumor cell sensitivity to conventional and novel anti-MM therapies, and thereby inform single agent or combination individualized therapy.

## MATERIALS AND METHODS

### Reagents

Thalidomide, lenalidomide, pomalidomide, bortezomib and carfilzomib were purchased by Selleck Chemicals LLC (Houston, TX, USA). Reserpine, doxorubicin, dexamethasone and melphalan were obtained from Sigma-Aldrich (St. Louis, MO, USA).

### Patient samples and cell cultures

Overall BM samples from 52 patients with premalignant condition or MM were studied including: smoldering MM (N=8), newly diagnosed MM (N=5), relapsed MM (N=23), and relapsed/refractory MM (N=16). Approval for these studies was obtained from the Dana-Farber Cancer Institute Institutional Review Board. All patient samples were collected after informed consent in accordance with the Declaration of Helsinki protocol. After BM aspiration (Figure 1A, i), fresh BM mononuclear cells (MNC) were obtained by ammonium chloride based-lyses of red cells (iii), followed by washing in phosphate-buffered saline (PBS). Cells were then cultured in complete α-MEM (Cellgro, Mediatech, VA, USA) supplemented with 5% platelets, 2 U/mL Heparin, 100 U/mL penicillin, 100 μg/mL streptomycin (Gibco, Grand Island, NY, USA), and maintained at 37°C in 5% CO_2_ to generate MSC (iv). After 3 days, the non-adherent fraction of MNCs from BM samples was collected and frozen, whereas the MSC (adherent fraction) were expanded with fresh complete α-MEM added every 3 days (Figure 1A, iv). In addition, MSC were cultured with respective lineage differentiation media, as described in Supplemental Methods. MM cell lines were obtained from American Type Culture Collection (ATCC; Manassas, VA, USA) and maintained in RPMI 1640 medium (Cellgro, Mediatech, VA, USA) supplemented with 10% heat-inactivated fetal bovine serum, 2mM L-glutamine, 100 U/mL penicillin, and 100 μg/mL streptomycin.

### Establishment of 2D and 3D cultures

The MSC (8×10^4^ cells/cm^2^) were seeded on plastic surface in complete Dulbecco's modified Eagle medium (DMEM; Cellgro, Mediatech, VA) supplemented with 10% heat-inactivated fetal bovine serum, 100 U/mL penicillin, 100 μg/mL streptomycin, and maintained at 37°C in 5% CO_2_ to establish monolayer (2D) cultures. For generation of 3D models, 0.5% PuraMatrix hydrogel (5mg/ml; BD Biosciences, San Jose, CA, USA) diluted in tissue culture-grade water was placed on the well surface. For gelation, complete DMEM medium was slowly and carefully added and incubated for 30min at 37°C in 5% CO_2_. After incubation, the medium was changed 2-3 times over a period of 1 hour to equilibrate the growth environment to physiological pH. MSC (8×10^4^ cells/cm^2^) were then seeded on the surface of the pH equilibrated hydrogel.

### Immuno-fluorescence/mass analyses

Cell surface phenotype of MSC was evaluated by 11-color multiparametric flow cytometry, as described in Supplemental Methods. Expression of PC phenotype was determined by flow cytometry analyses. Immune subpopulations were detected by mass cytometry (CyTOF) analyses of 23 cell surface and 2 intracellular phenotypic markers, as described in Supplemental Methods. The expression of ECM molecules, integrins, and other key niche molecules listed in Supplemental Methods was evaluated by immunofluorescence staining, using multiparametric flow cytometry and confocal microscopy. Multiplex fluorescence bead-based cytokine immunoassays were performed for quantification of multiple cytokine, chemokine, growth factor, and cancer biomarkers, as described in Supplemental Methods. MM cell proliferation and drug resistance in co-culture systems were evaluated with CFSE and Annexin V-PE apoptosis assays using flow cytometry. Functional assays to identify SP fractions were detected be Hoechst 33342 dye staining, as described in Supplemental Methods.

### Molecular profiling analyses

Gene expression of MSC was determined using Human Gene 1.0 ST array analyses, as described in Supplemental Methods. Reverse transcription polymerase chain reaction (RT-PCR) was performed using Tagman assays, as described in Supplemental Methods.

### Statistical analyses

Statistical significance of two groups was determined by parametric-paired t-test (mean comparison) and non-parametric-Wilcoxon signed rank test (median comparison). The differences in the mean and median values among three and more paired groups were tested using one way repeated measures (RM) analysis of variance (ANOVA) and RM ANOVA on ranks (Friedman test), respectively. A multiple comparison procedures, the Holm-Sidak method and the Tukey test, were used to isolate the group or groups that differ from the others. A nonparametric Kruskal-Wallis ANOVA on ranks was used for comparison of unpaired samples. In all statistical tests, p values <0.05 were considered significant.

## SUPPLEMENTARY MATERIALS METHODS AND FIGURES


