# Supplementary material for: A novel 3D mesenchymal stem cell model of the multiple myeloma bone marrow niche: biologic and clinical applications
Source: Oncotarget. 2016 Oct 13;7(47):77326–41. doi: 10.18632/oncotarget.12643 (PMC5357212; doi:10.18632/oncotarget.12643)
Supplement: Supplementary file 1 [file oncotarget-07-77326-s001.pdf]

A novel 3D mesenchymal stem cell model of the multiple myeloma bone marrow niche: biologic and clinical applications

SUPPLEMENTARY METHODS

Multiparametric immunophenotypic analysis

Erythrocyte-removed BM cells and MSC were washed with phosphate-buffered saline (PBS) and stained with antibody mix cocktail in PBS containing 2% bovine serum albumin (BSA; Sigma-Aldrich, St. Louis, MO, USA) for 30 min at 4°C. Phenotypic analyses of MSC were performed using a single 11-color combination of monoclonal antibodies containing Human lineage cocktail (CD2, CD3, CD14, CD16, CD19, CD56, CD235a)-FITC/CD166-PerCP-Cy5.5/CD271-PE/HLA-ABC-PE-Cy5/CD73-PE-Cy7/CD105-Alexa Flour 647/CD90-Alexa Flour 700/n-IR-viability dye-APC-Cy7/CD146-V450/CD45-V500/HLA-DR-eFluor605NC (BioLegend, San Diego, CA, USA; BD Biosciences, San Jose, CA, USA). Plasma cells in BM MNC were identified by CD138-PE and CD38-APC staining, and expression of CXCR4-PerCP-Cy5.5 on plasma cells was determined (BD Biosciences, San Jose, CA, USA). After incubation, cells were washed twice in PBS containing 2% BSA, and then resuspended in PBS for analysis on a Fortessa cytometer (Becton Dickinson, Mountain View, CA).

Mass cytometry analysis

Phenotypic analysis of MNC from MM patients (N=5) before and after co-culture with allogeneic MSC in 3D versus 2D conditions was performed using mass cytometry (CyTOF) technology. Briefly, cells were fixed with formaldehyde (PFA; Electron Microscopy Sciences, Hatfield, PA, USA) added directly to growth media at a final concentration of 1.6% for 10 minutes at room temperature (RT). Cells were collected and washed with cell staining media (CSM; PBS with 0.5% BSA, 0.02% sodium azide). Before staining with surface markers antibodies, cells were incubated with 5 µl of the Fc-receptor blocking solution for 10 minutes at RT to block non-specific binding (BioLegend, San Diego, CA, USA), and then with cell surface antibody cocktail to 100 µl final volume for 30 min at RT. Antibody cell surface mix cocktail contained: HLA-DR-141Pr, CD19-142Nd, CD3-143Nd, CD15-144Nd, CD11b-146Nd, CD4-147Sm, CD33-148Nd, CD8-149Sm, CD13-150Nd, CD38-152Sm, CD7-153Eu, CD73-155Gd, CD45-154Sm, CD14-158Gd, CD11c-159Tb, CD45-RA-160Gd, CD90-161Dy, CD45-RO-162Dy, CD63-163Dy, CD16-171Yb, CD138-172Yb, CD105-173Yb, and CD56-175Lu (BioLegend, San Diego, CA, USA; BD Biosciences, San Jose, CA, USA). Antibodies were conjugated with the MaxPAR antibody conjugation kit with respective mass isotopes (Fluidigm, San Francisco, CA, USA), according to the manufacturer's instructions. After staining, cells were washed twice with CSM and permeabilized with 4°C methanol for 10 min at 4°C, then stored at -80°C. Cells were washed twice in CSM to remove remaining methanol, and then stained with intracellular antibody cocktail (cyto kappa-165Ho and cyto lambda-174Yb) in 100 µl for 1 hour at RT. Cells were washed twice in CSM and stained with 1 mL of 1:2000 191/193Ir DNA intercalator (Fluidigm, San Francisco, CA, USA) diluted in PBS with 1.6% PFA for 20 min at RT. Cells were washed with PBS alone and then with dH2O. Cells (10^6 cells per ml) were analysed on a CyTOF II mass cytometer (Fluidigm, San Francisco, CA, USA). All gating strategies and extraction of median expression level were defined using Cytobank software (Mountain View, CA, USA). High-dimensional data were analysed by spanning-tree progression analysis of density-normalized events (SPADE), using all 23 cell surface markers as clustering markers. All samples with all conditions were normalized by beads and analysed by SPADE simultaneously, so the resulting tree structure would capture all subpopulations present in the entire dataset. Clusters in SPADE tree were grouped manually and annotated into immunophenotypic populations, based upon examination of positive expression of relevant biaxial plots of the cell events in each cluster.

Flow cytometry immunofluorescence analysis

Antibody (1 µg) was incubated with 5 µl of the Zenon IgG labelling reagent (Alexa405; Alexa488; R-PE; Alexa647; Molecular Probes, Eugene, OR, USA) for 5 min at RT. Then, 5 µl of the Zenon blocking reagent was added to the reaction mixture and incubated for 5 min at RT. MSCs were washed with PBS containing 2% BSA, and then stained with antibody mix cocktail for 30 min at 4°C. Antibody mix cocktails to stain MSC contained: N-cadherin-PE or nestin-PE/integrin α4-APC; integrin β1-Alexa 405/vitronectin-FITC/CXCL12-APC; laminin-Alexa405/fibronectin-Alexa 488/collagen I-PE/collagen IV-Alexa647; and integrin α5β1-Alexa488/integrin αvβ3-PE/integrin α2β1-Alexa647 (BioLegend, San Diego, CA, USA; Sigma-Aldrich, St. Louis, MO, USA). After staining, cells were washed in PBS containing 2% BSA, and then analysed on a Fortessa cytometer (Becton Dickinson, Mountain View, CA).

Confocal immunofluorescence analysis

The cultures set on coverslips placed in 6-well plates were fixed with 2% paraformaldehyde for 20 min at RT and permeabilized with 0.5% Triton X-100 in PBS for 10 min at 4°C. They were then, rinsed 3 times with PBS-Glycine for 10min each and incubated with blocking PBS supplemented with 1% BSA, 2% Triton X-100, 0.4% Tween-20 and 10% goat serum for 1 hour. After blocking, cells were incubated with primary antibody directed at fibronectin, laminin, vitronectin, collagen I and collagen IV, α4 integrin, β1 integrin, integrin α2β1 or integrin α5β1 (Sigma-Aldrich, St. Louis, MO, USA) in blocking PBS at 4°C overnight, and then with fluorescence-tagged secondary antibody (goat anti-mouse IgG, IgM secondary antibody; Molecular Probes, Eugene, OR, USA) for 1 hour at RT in dark. After several washings with PBS, cells were stained with DAPI (1μg/ml; Molecular Probes, Eugene, OR, USA) in PBS for 20 min at RT to demarcate the nuclei. The control cells were stained with secondary antibody alone to detect non-specific interaction. Similarly, the hydrogel was tested for non-specific bindings with antibodies and for interference with washing steps. Imaging was done using confocal laser-scanning microscopy on an inverted Leica DMI6000 with a motorized X, Y stage and piezo Z stepper using Leica LAS AF Lite software (Leica Microsystems Inc., Buffalo Grove, IL, USA). The images were captured in sequential mode. Serial optical sections of 0.5 microns were taken. Image analysis was done using Leica Application Suite (Leica Microsystems Inc., Buffalo Grove, IL, USA).

Lineage differentiation assays

MSC were cultured in 2D versus 3D system with respective lineage differentiation media (Life Technologies, Carlsbad, CA, USA) towards osteogenic lineage for 21 days, and adipogenic lineage for 14 days. Cultures were fed every third day. After differentiation, cells were fixed in 10% buffered formaldehyde for 20 min, and then washed twice with distilled water. Cells were stained with Oil Red O solution (Sigma-Aldrich, St. Louis, MO, USA) for 20 min to detect adipocytes or with 2% Alizarin Red (Sigma-Aldrich, St. Louis, MO, USA) for 30 min to detect osteoblasts. After respective lineage differentiation staining, cells were washed three times with distilled water and imaged on an inverted phase contrast Leica microscope, with a Leica DFC300Fx camera using 10X objective and Leica IM50 image-acquisition software Version 4 (Leica Microsystems Inc., Buffalo Grove, IL, USA).

Multiplex microbead-based cytokine immunoassays

Supernatants from 2D and 3D MSC were collected, filtered through 0.22 μm pore size filter, and stored at –80°C until analysis. Cytokine 27-Plex panel I included: interleukin (IL)-1β, IL-1 receptor antagonist (IL-1ra), IL-2, IL-4, IL-5, IL-6, IL-7, IL-8, IL-9, IL-10, IL-12(p70), IL-13, IL-15, IL-17, eotaxin, fibroblast growth factor (FGF) basic, granulocyte-colony stimulating factor (G-CSF), granulocyte-macrophage colony stimulating factor (GM-CSF), interferon (IFN)-γ, interferon-gamma induced protein (IP-10), monocyte chemotactic protein (MCP)-1, macrophage inflammatory protein (MIP)-1α, MIP-1β, platelet-derived growth factor (PDGF)-bb, regulated upon activation normal T-cell expressed and secreted (RANTES), tumor necrosis factor (TNF)-α, and vascular endothelial growth factor (VEGF) was performed according to the manufacturer's instruction manual in 96-well filter microplates. Similarly, a customized Cancer Biomarker panel included: soluble epidermal growth factor receptor (sEGFR), human epidermal growth factor receptor 2 (sHER2 neu), hepatocyte growth factor/scatter factor (HGF), leptin, osteopontin (OPN), stem cell factor (SCF), soluble Tie-2 receptor (sTie-2), angiopoietin-2 (Ang-2) and insulin-like growth factor-binding protein 1 (IGFBP-1). These assays are multiplex immunoassays built on fluorescent beads for the quantification of multiple cytokine, chemokine, growth factor, and cancer biomarkers. Briefly, appropriate cytokine standards and supernatants (50 μl) were incubated with the fluorescent dyed microspheres coupled to specific monoclonal antibody (50 μl), according to specific panel, for 30 min to 1 hour in the dark on a plate shaker. After washing steps, the beads were further incubated with the biotinylated detection antibody cocktail (25 μl/well) for 30 min at RT on a plate shaker, and then streptavidin-PE (50 μl/well) was added for 10 min. Finally, assay buffer (125 μl) was added to each well, and the plate was analyzed with the Bio-Plex Suspension Array System (Bio-Rad Laboratories, Philadelphia, PA, USA). The analyte concentration was calculated using Bio-Plex Manager software provided by the manufacturer.

CFSE assay

To distinguish MM cells (MM cell lines and BM cells from primary patients) in co-culture models, MM cells were labeled with carboxyfluorescein diacetate succinimidyl ester (CFSE) fluorescent cytoplasmic dye, and seeded on the unlabeled 2D and 3D MSC after 5 days in culture. Changes in mean fluorescence intensity of CFSE signal were used as a surrogate marker of cell proliferation. Briefly, MM cells were labeled with 0.25 μM CFSE (Molecular Probes, Eugene, OR, USA) for 10 minutes at 37°C in serum-free RPMI in the dark. The reaction was stopped by adding RPMI 1640 medium supplemented with 2% FBS, and cells were washed 3 times with 10% FBS RMPI 1640. Cells were then seeded in 2D or 3D models with unlabeled MSC seeded 5 days earlier, followed by drug-based studies.

Apoptosis assay

Apoptotic cells were quantified using the Annexin V-PE apoptosis assay. Briefly, both suspension and adherent cells were collected and washed with cold PBS. Cells were resuspended in 100 µl of manufacturer-supplied 1X binding buffer and mixed with 5 µl of Annexin V-PE (BD Biosciences, San Jose, CA, USA) and 5 µl of 7-amino-actinomycin D (7-AAD; Molecular Probes, Eugene, OR, USA). After 15 min incubation in the dark at RT, cells were analysed by a FACS Canto II flow cytometer (Becton Dickinson, Mountain View, CA) using a 96-well format.

Side population analysis

The cells were labelled with Hoechst 33342 dye using the methods described by Goodell et al with modifications. The cells were washed in pre-warmed RPMI with 2% FBS and 10 mmol/L Hepes buffer (Life Technologies, Carlsbad, CA, USA), and then resuspended in RPMI with 2% FBS and 10 mmol/L Hepes buffer containing 5 µg/mL of Hoechst 33342 dye (Molecular probes, Eugene, OR); cells were then incubated for 90 min at 37 °C with intermittent shaking. As a negative control, cells were preincubated with 50 µmol/L reserpine, an ABC transporter inhibitor. At the end of the incubation, cells were washed with ice-cold PBS containing 2% FBS and 10 mmol/L Hepes buffer. To gate only viable cells 7-AAD (Molecular probes, Eugene, OR, USA; final concentration = 1 µg/mL) in PBS with 2% FBS and 10 mmol/L Hepes buffer was added to the cells. The cells were analyzed by a FACS Aria Special Sorter equipped with UV laser (Becton Dickinson, Mountain View, CA, USA). The Hoechst 33342 dye was excited at 357 nm, followed by dual-wavelength fluorescence analysis (blue, 402-446 nm; 650-670 nm).

Gene expression and reverse transcription polymerase chain reaction analysis

RNA of MSC was extracted using Trizol Reagent (Life technologies, Carlsbad, CA, USA) and quantified by a Nanodrop spectrophotometer (Labtech International Ltd, East Sussex, TN, USA). Briefly, cells were collected in 1mL Trizol Reagent, and then chloroform was added. RNA was precipitated with isopropyl alcohol and then with 75% ethanol, and resuspended in Nuclease Free-water. After quantification, 1000ng of RNA was used to synthesize cDNA via the Superscript II First strand synthesis Kit (Life technologies, Carlsbad, CA, USA), according to the manufacturer's instructions. Gene expression analyses were determined using Human Gene 1.0 ST array system (Affymetrix, Santa Clara, CA, USA). Statistical analyses were performed using GeneSpring software (Agilent Technologies, Santa Clara, CA, USA). CEL files contained a roster of intensity values of individual probes and were imported into GeneSpring to analyze gene expression changes. Significant differences (fold of expression change ≥ 2.0) in gene expression were evaluated for every pair of samples separately (non-averaged). Pathway analysis was performed to reveal molecular pathways significantly altered in 2D versus 3D MSC ($p \leq 0.05$). Hierarchical cluster analyses based on entities and conditions using normalized intensity values were performed using GeneSpring software. Reverse transcription polymerase chain reactions (RT-PCR) were performed using Tagman Gene Expression Assays (Life technologies, Carlsbad, USA). cDNAs were diluted 1:100 and amplified in a 10 uL reaction. Primers (MGP (Hs00179899_m1), RUNX2 (Hs01047973_m1), ADAMTS4 (Hs00192708_m1), MMP13 (Hs00233992_m1), SPP1 (Hs00959010_m1), CALM1 (Hs00300085_s1), and FGFR1 (Hs00915142_m1)) were used at concentration of 200 nmol. GAPDH (Hs02758991_g1) was used as a loading control. Thermal cycling conditions were: 2 minutes at 50°C, 10 minutes at 95°C, 40 cycles at 95°C for 15 seconds, followed by 1 minute at 60°C. RT-PCR was performed using Bio-Rad CFX96™ Real-Time PCR Detection system (Bio-Rad Laboratories, Philadelphia, PA, USA) and a normalized fold expression was calculated with the ΔΔCq method by Bio-Rad Software Manager, Version 1.6, provided by the manufacturer (Bio-Rad Laboratories, Philadelphia, PA, USA).

SUPPLEMENTARY FIGURES

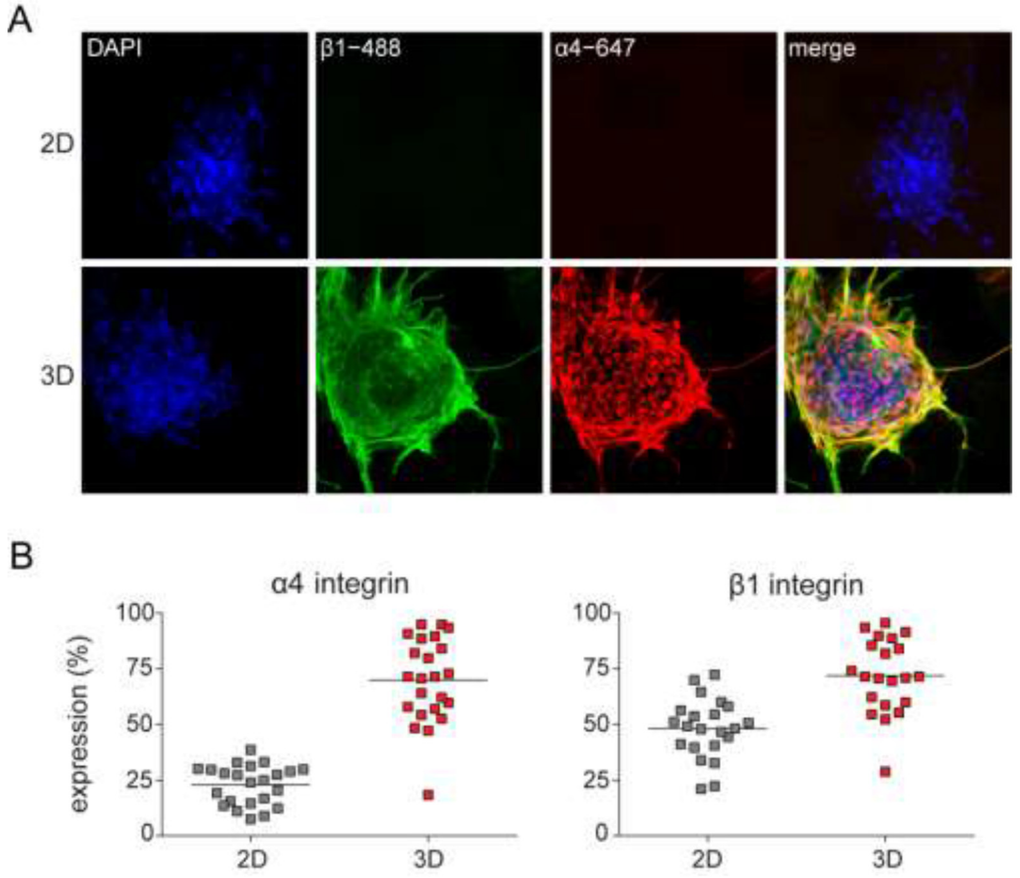

Supplementary Figure S1: β1 and α4 integrin subunits in 3D vs 2D models. A. Co-localization (dimer formation, yellow color) of β1 (green) and α4 (red) integrin subunits assessed using confocal microscopy. Nuclei are identified by DAPI staining (blue). **B.** Flow cytometry analysis of β1 and α4 subunits in 3D compared to monolayer 2D MSC. Each block represents MSCs from one MM patient.

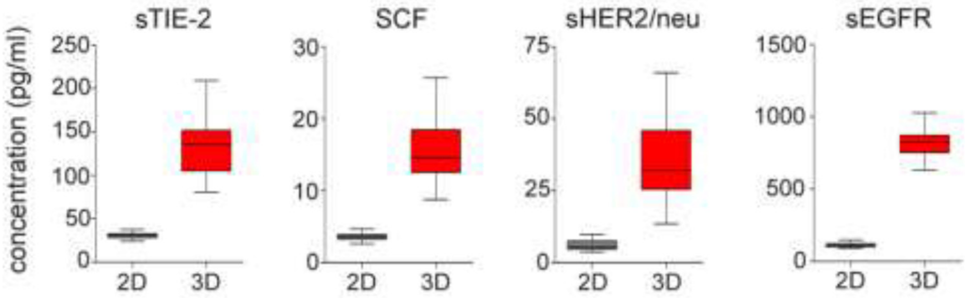

Supplementary Figure S2: Production of Tie-2, SCF, sHER2neu, and sEGFR in 3D vs 2D models. Production of soluble Tie-2 receptor (sTie-2), stem cell factor (SCF), human epidermal growth factor receptor 2 (sHER2neu), and soluble epidermal growth factor receptor (sEGFR) secreted by MSC in 2D vs 3D conditions after 5 days was analyzed by multiplex luminex technology.

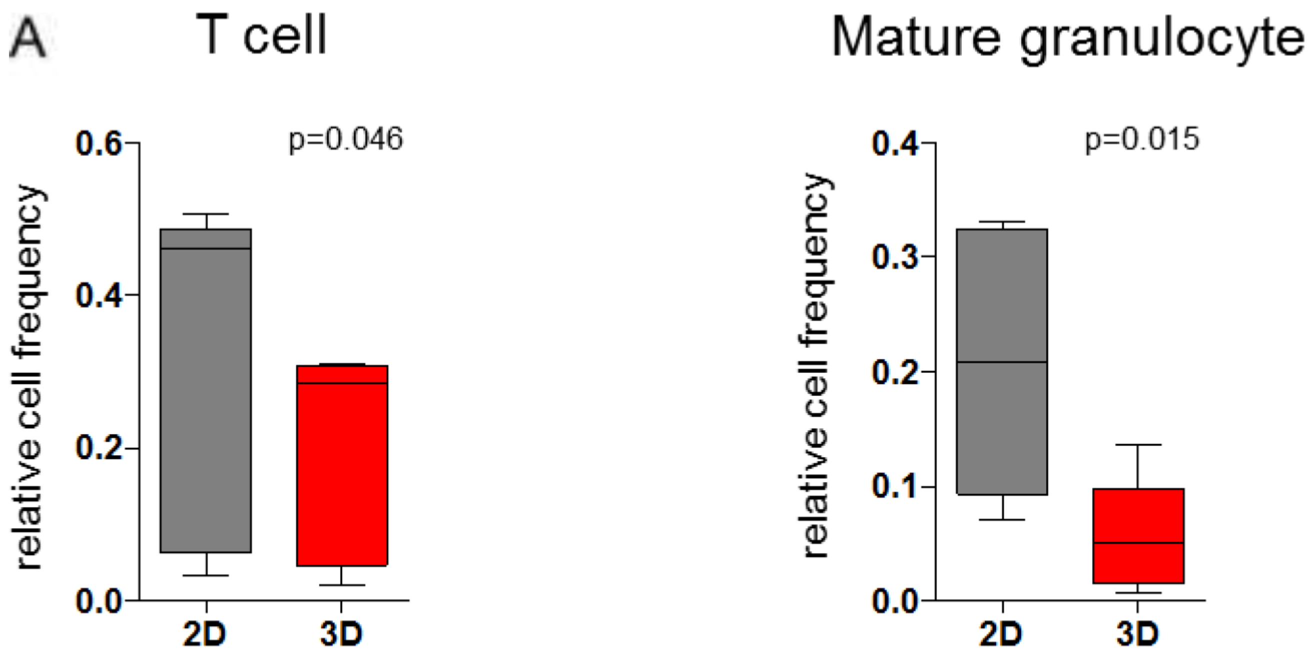

Supplementary Figure S3: Immune cell subsets in 3D vs 2D models by SPADE analysis. A. Cell frequencies of T cell and mature granulocyte populations of 5 MM MNC co-culture with allogeneic MSC in 2D and 3D model analysed by SPADE. (*Continued*)

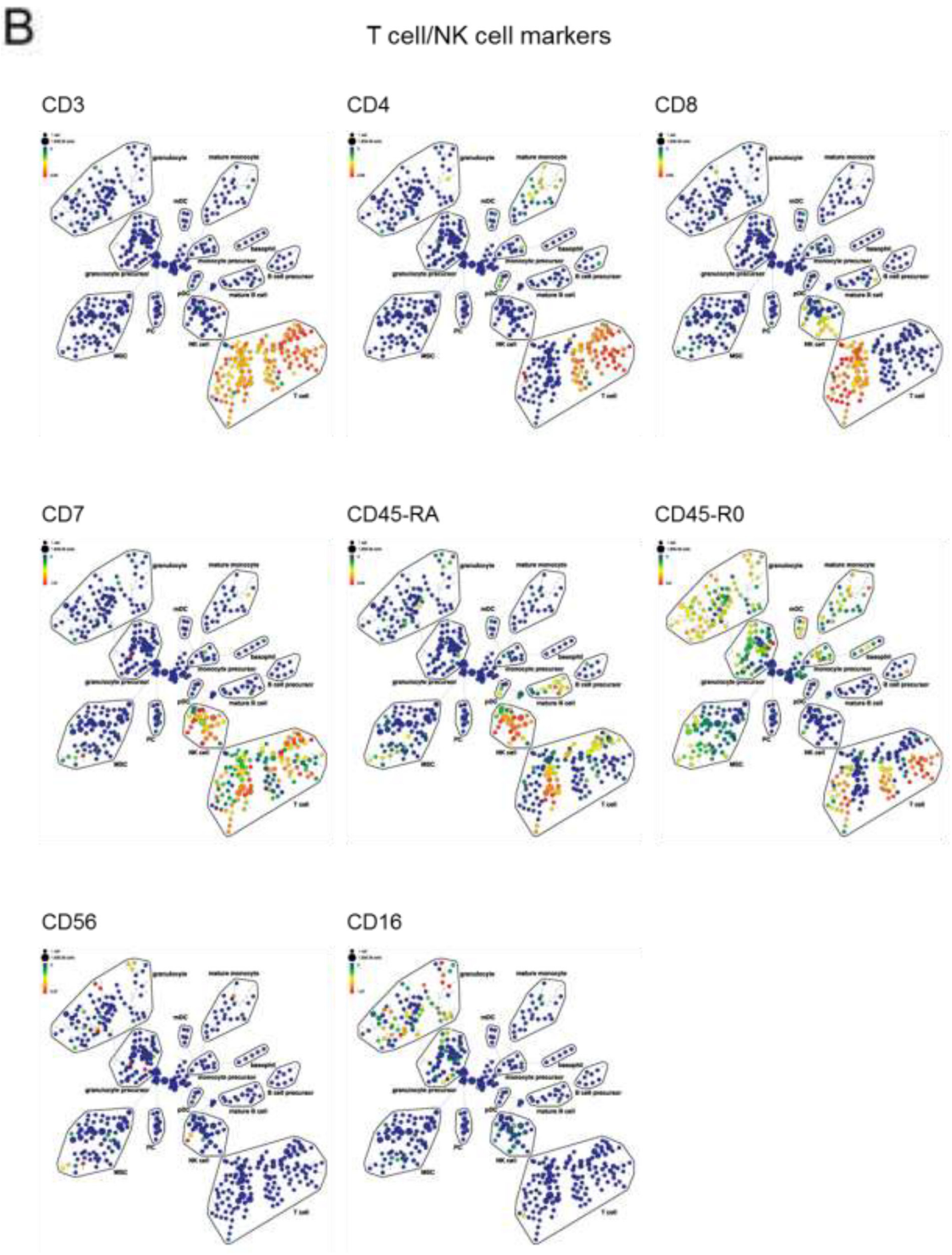

Supplementary Figure S3: (*Continued*) Immune cell subsets in 3D vs 2D models by SPADE analysis. B. SPADE plots of bone marrow MNC of representative MM patient co-cultured with allogeneic MSC in 3D model. SPADE clustering was performed on all samples (N=5; at conditions before co-culture and then co-cultured in 3D or 2D models) simultaneously to generate a single tree structure for all samples, with MNC before co-culture used as baseline for both 2D and 3D co-culture models. All cell events from each sample were then mapped to the common tree structure. SPADE organized cells to cluster in the tree by a hierarchy of related phenotype. The size of each node is correlated to the fraction of cells mapping to the node. Each node of the SPADE tree is colored to median expression of the indicated markers, from low (blue color) to high (red color) expression. Unsupervised clustering analyses define the main immunophenotypic populations in SPADE tree, based on selected clustering cell surface markers. Immunophenotypic subpopulations were identified by grouping of nodes on the basis of the median marker expression level of each node, and analysis of the relevant biaxial plots. Clustering of 23 surface markers and 2 intracellular markers, organized according to median expression by SPADE plots and relative median phenotypic expression in specific immune subpopulations are shown by box and whisker plots (median signal intensity and interquartile range) in 2D vs 3D co-culture models: T cell/NK cell markers (CD3, CD4, CD8, CD7, CD45-RA, CD45-RO, CD56 and CD16). (*Continued*)

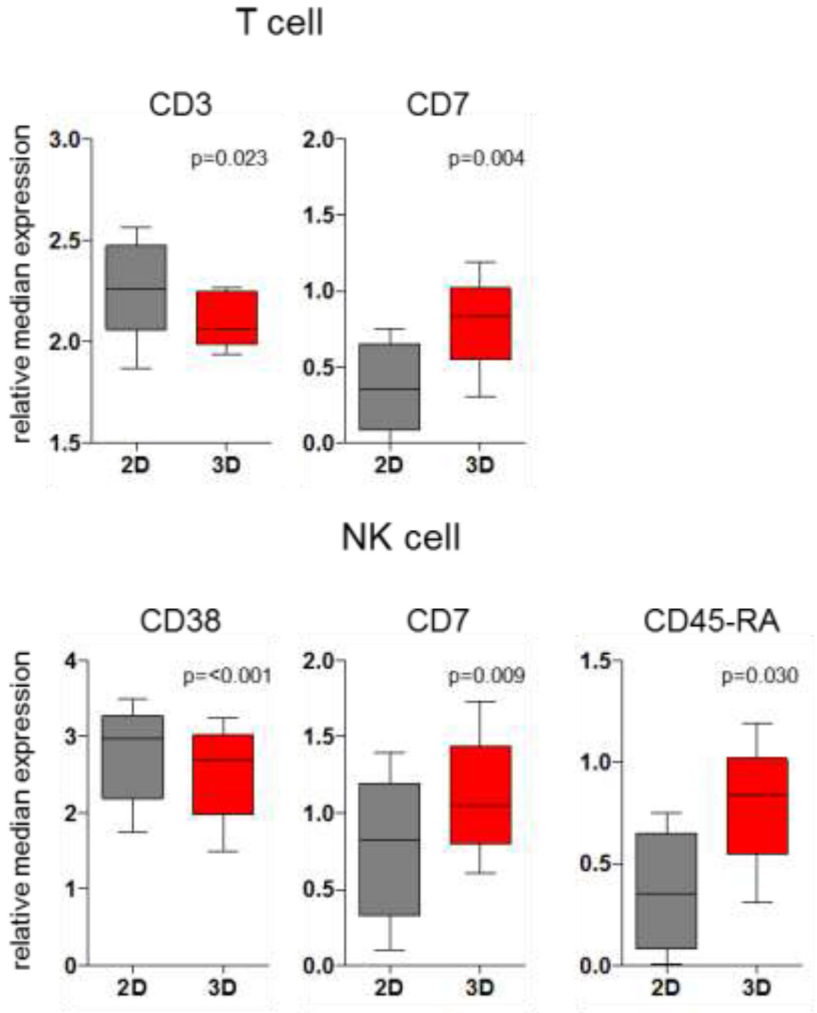

Supplementary Figure S3: (*Continued*) Immune cell subsets in 3D vs 2D models by SPADE analysis. B. SPADE plots of bone marrow MNC of representative MM patient co-cultured with allogeneic MSC in 3D model. SPADE clustering was performed on all samples (N=5; at conditions before co-culture and then co-cultured in 3D or 2D models) simultaneously to generate a single tree structure for all samples, with MNC before co-culture used as baseline for both 2D and 3D co-culture models. All cell events from each sample were then mapped to the common tree structure. SPADE organized cells to cluster in the tree by a hierarchy of related phenotype. The size of each node is correlated to the fraction of cells mapping to the node. Each node of the SPADE tree is colored to median expression of the indicated markers, from low (blue color) to high (red color) expression. Unsupervised clustering analyses define the main immunophenotypic populations in SPADE tree, based on selected clustering cell surface markers. Immunophenotypic subpopulations were identified by grouping of nodes on the basis of the median marker expression level of each node, and analysis of the relevant biaxial plots. Clustering of 23 surface markers and 2 intracellular markers, organized according to median expression by SPADE plots and relative median phenotypic expression in specific immune subpopulations are shown by box and whisker plots (median signal intensity and interquartile range) in 2D vs 3D co-culture models: T cell/NK cell markers (CD3, CD4, CD8, CD7, CD45-RA, CD45-RO, CD56 and CD16). (*Continued*)

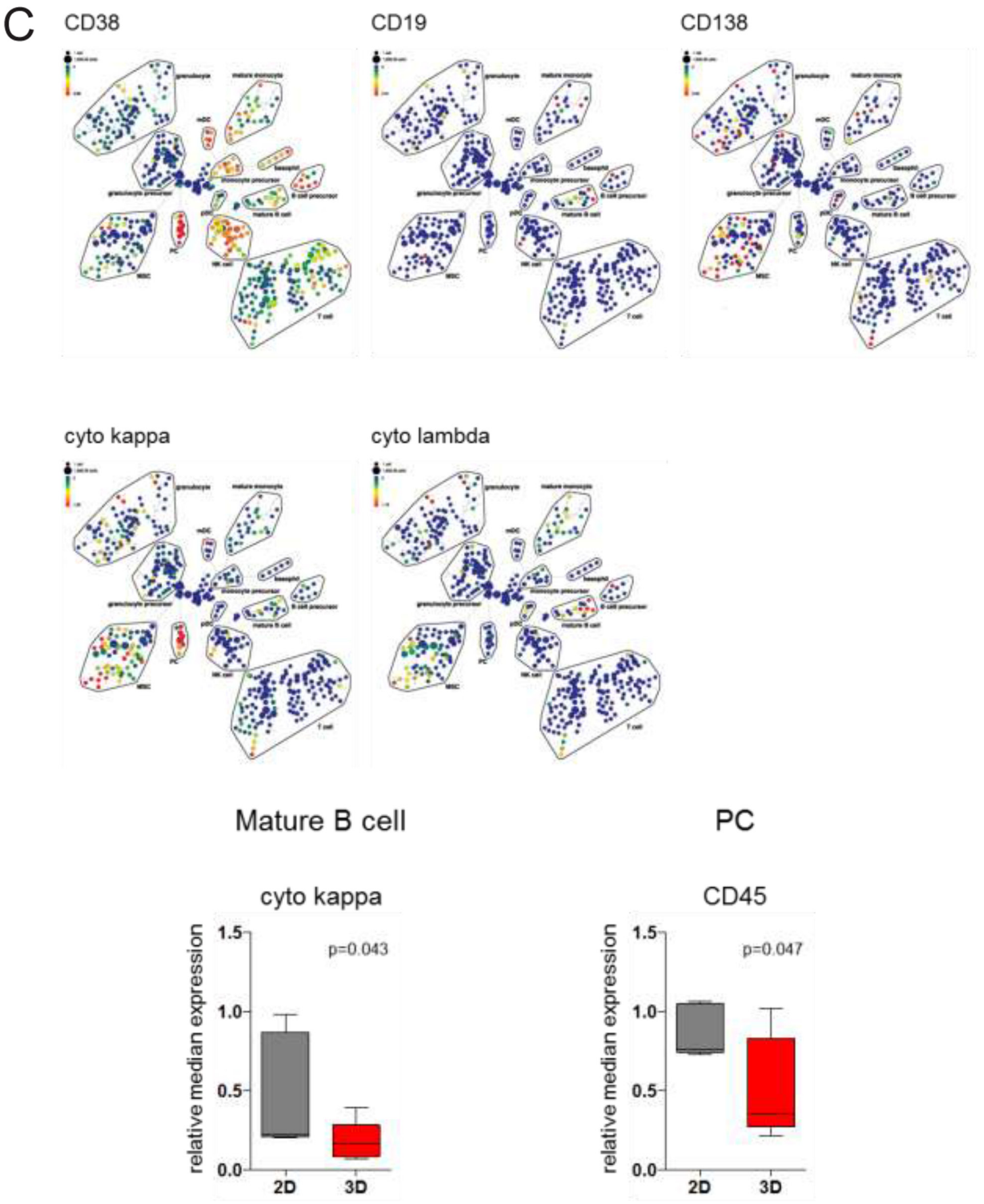

Supplementary Figure S3: (*Continued*) Immune cell subsets in 3D vs 2D models by SPADE analysis. C. B cell/PC markers (CD38, CD19, CD138, cyto kappa and cyto lambda); (*Continued*)

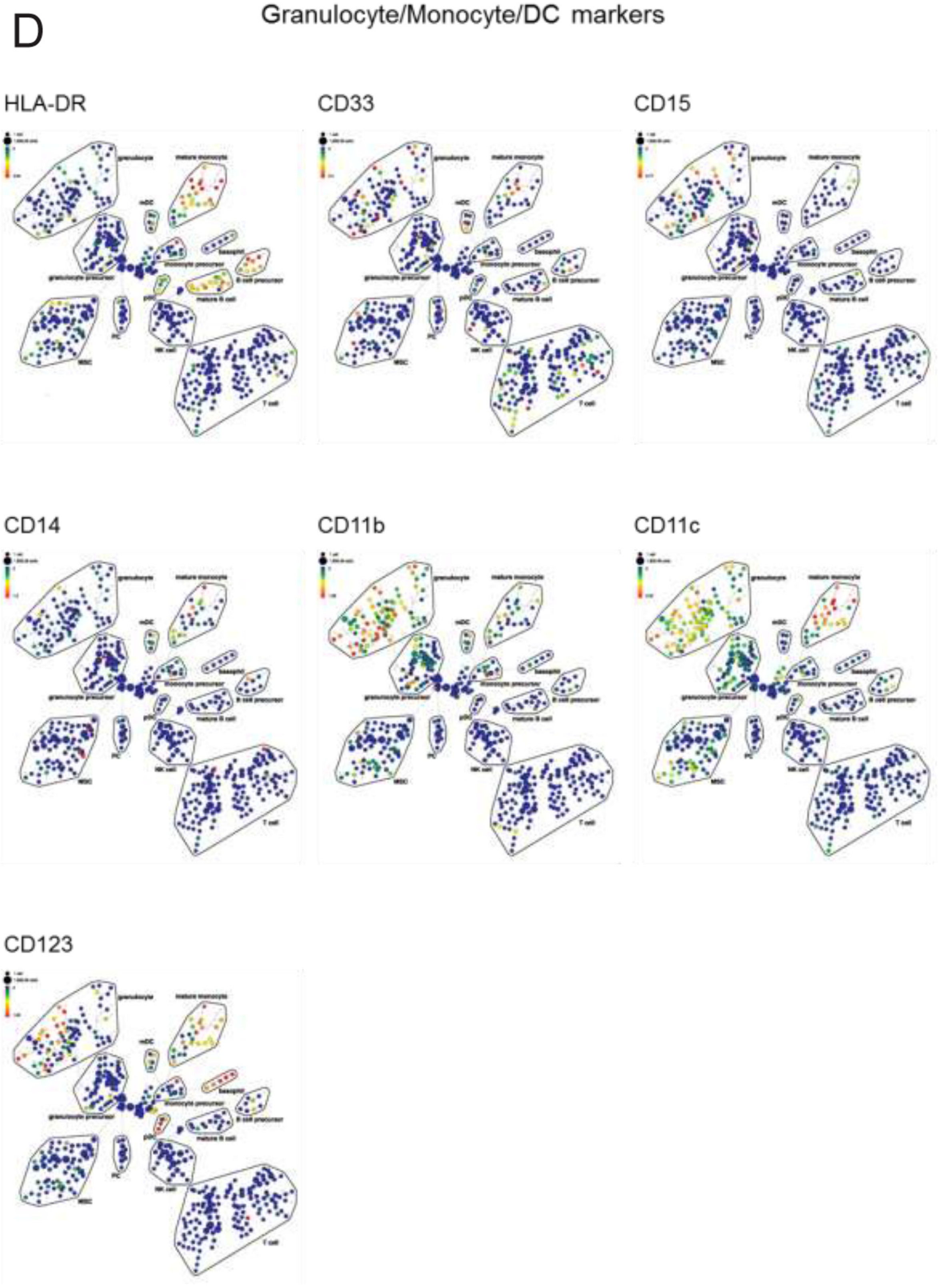

Supplementary Figure S3: (*Continued*) Immune cell subsets in 3D vs 2D models by SPADE analysis. D. Granulocyte/Monocyte/DC markers (HLA-DR, CD33, CD15, CD14, CD11b, CD11c and CD123); (*Continued*)

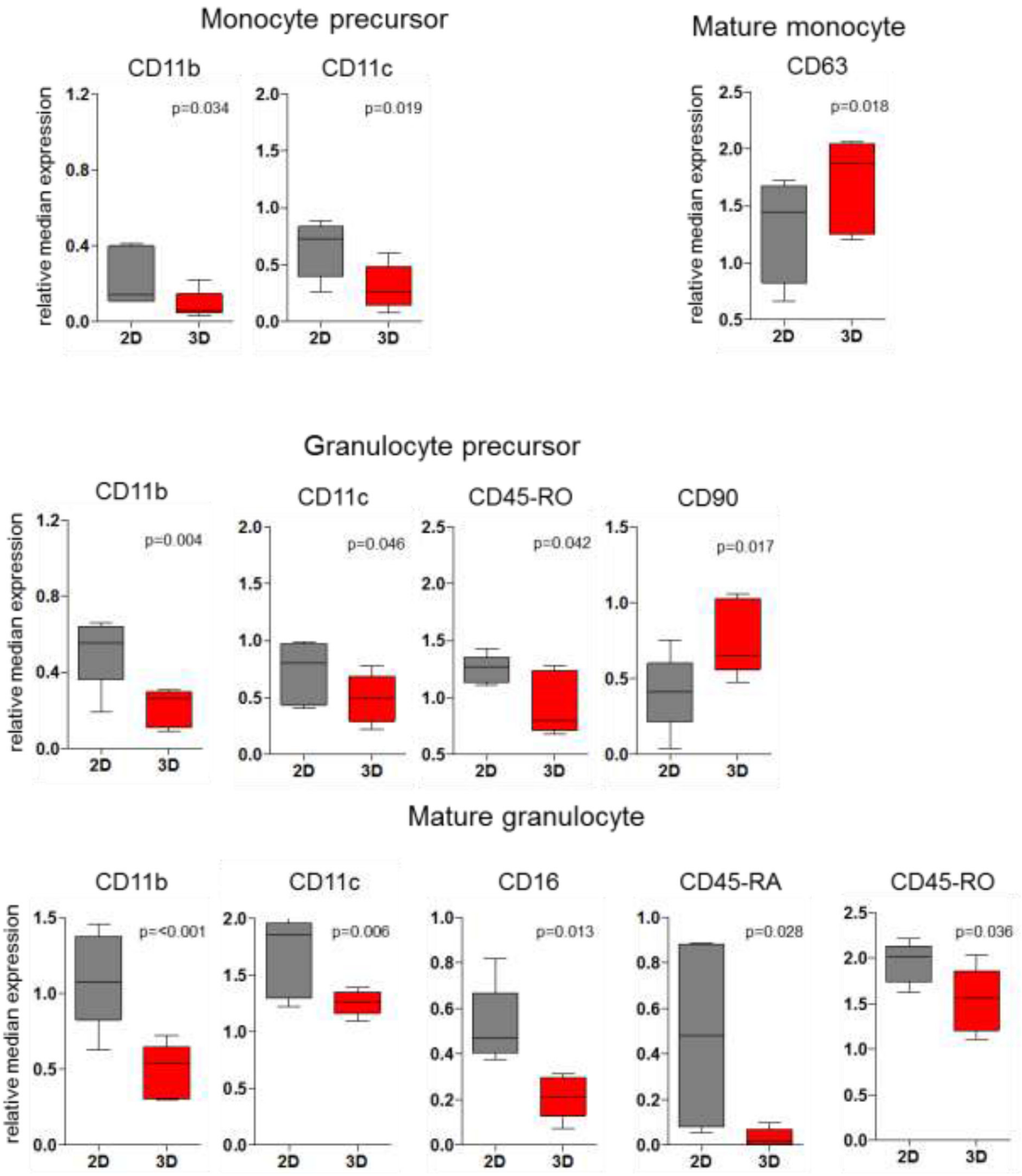

Supplementary Figure S3: (*Continued*) Immune cell subsets in 3D vs 2D models by SPADE analysis. D. Granulocyte/Monocyte/DC markers (HLA-DR, CD33, CD15, CD14, CD11b, CD11c and CD123); (*Continued*)

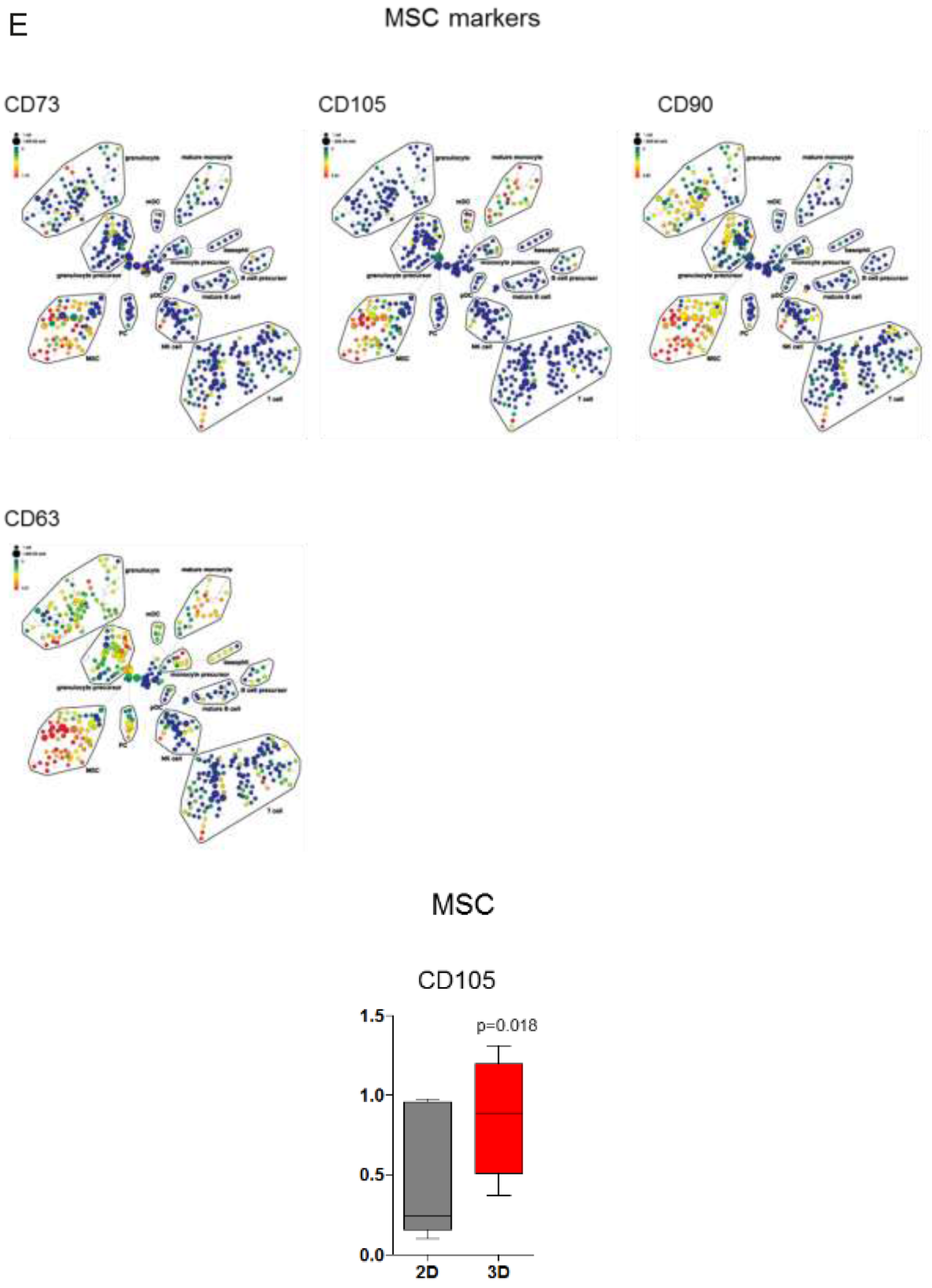

Supplementary Figure S3: (*Continued*) Immune cell subsets in 3D vs 2D models by SPADE analysis. E. MSC markers (CD73, CD105, CD90 and CD63) (Holm-Sidak of only $p < 0.05$ values).

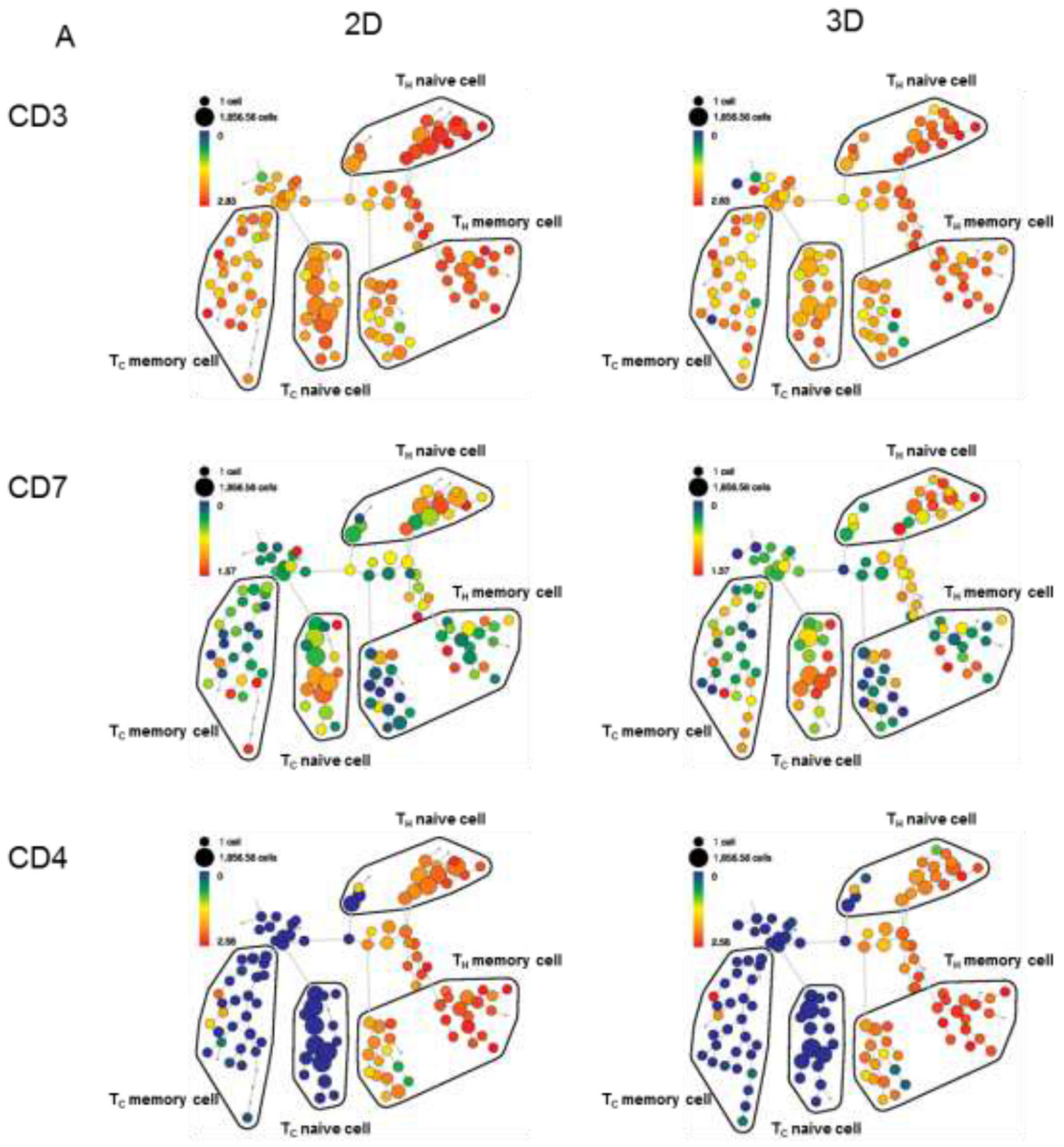

Supplementary. Figure. 4: T cell subpopulations in 3D vs 2D models by SPADE analysis. SPADE plots of immune T cell subpopulations of representative MM patient MNC co-cultured with allogeneic MSC in 2D or 3D models. SPADE clustering was performed on all samples (N=5; at conditions before co-culture, and then co-cultured in 3D or 2D models) simultaneously to generate a single tree structure for all samples, with MNC before co-culture used as baseline for both 2D and 3D co-culture models. T cells were clustered based on T specific cluster markers into 4 T cell subpopulations: TH (helper) naïve cell (CD3+CD4+CD45-RA+), TH memory cell (CD3+CD4+CD45-RO+), TC (cytotoxic) naïve cell (CD3+CD8+CD45-RA+), and TC memory cell (CD3+CD8+CD45-RO+). The median expression of T cell specific immunophenotypic markers (CD3; CD7; CD4; CD8; CD45-RA and CD45-RO) in 2D and 3D co-culture conditions is shown.

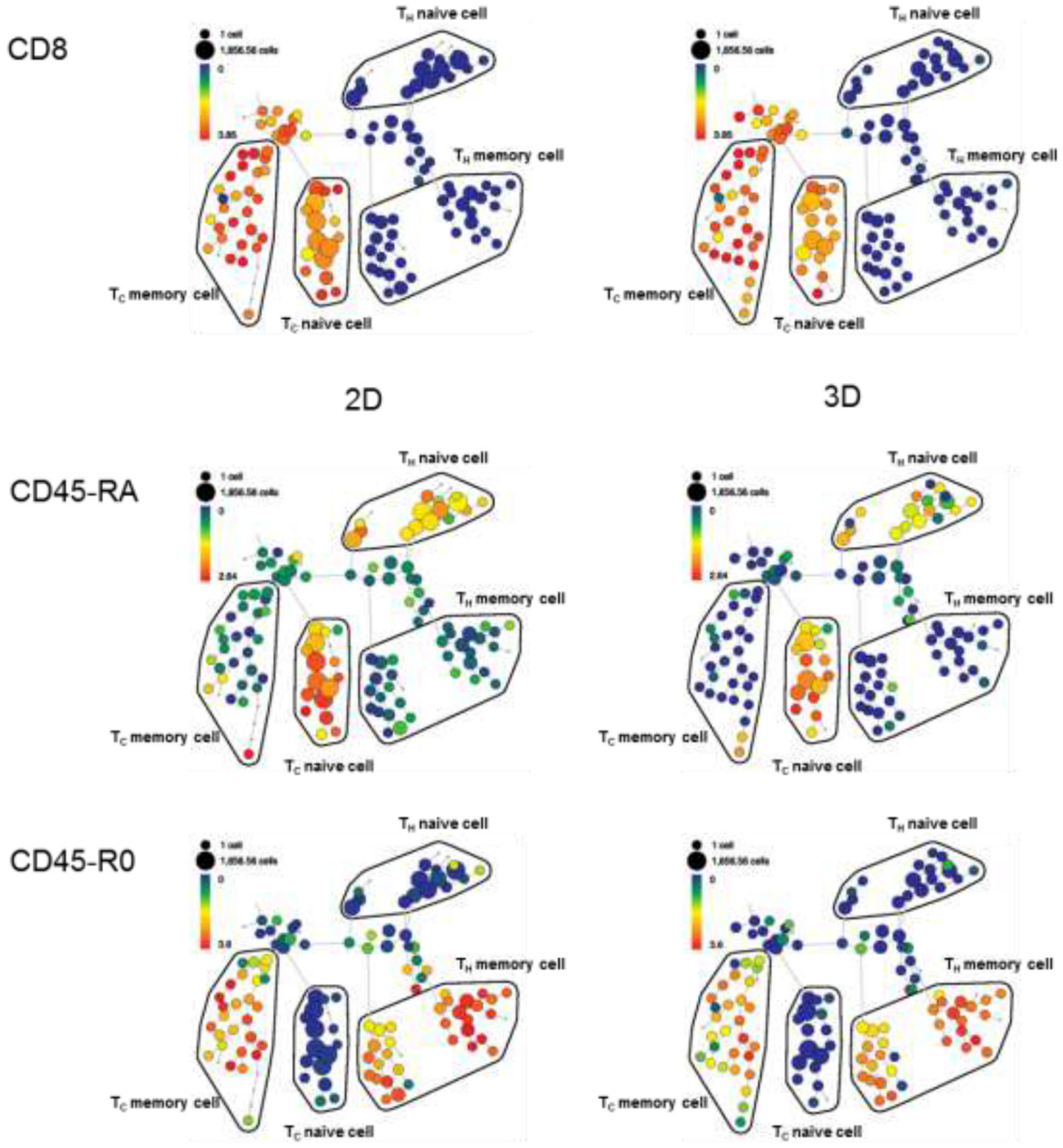

Supplementary Figure S4: (*continued*) T cell subpopulations in 3D vs 2D models by SPADE analysis. A. SPADE plots of immune T cell subpopulations of representative MM patient MNC co-cultured with allogeneic MSC in 2D or 3D models. SPADE clustering was performed on all samples (N=5; at conditions before co-culture, and then co-cultured in 3D or 2D models) simultaneously to generate a single tree structure for all samples, with MNC before co-culture used as baseline for both 2D and 3D co-culture models. T cells were clustered based on T specific cluster markers into 4 T cell subpopulations: T_H (helper) naïve cell (CD3+CD4+CD45-RA+), T_H memory cell (CD3+CD4+CD45-RO+), T_C (cytotoxic) naïve cell (CD3+CD8+CD45-RA+), and T_C memory cell (CD3+CD8+CD45-RO+). The median expression of T cell specific immunophenotypic markers (CD3; CD7; CD4; CD8; CD45-RA and CD45-RO) in 2D and 3D co-culture conditions is shown. (*continued*)

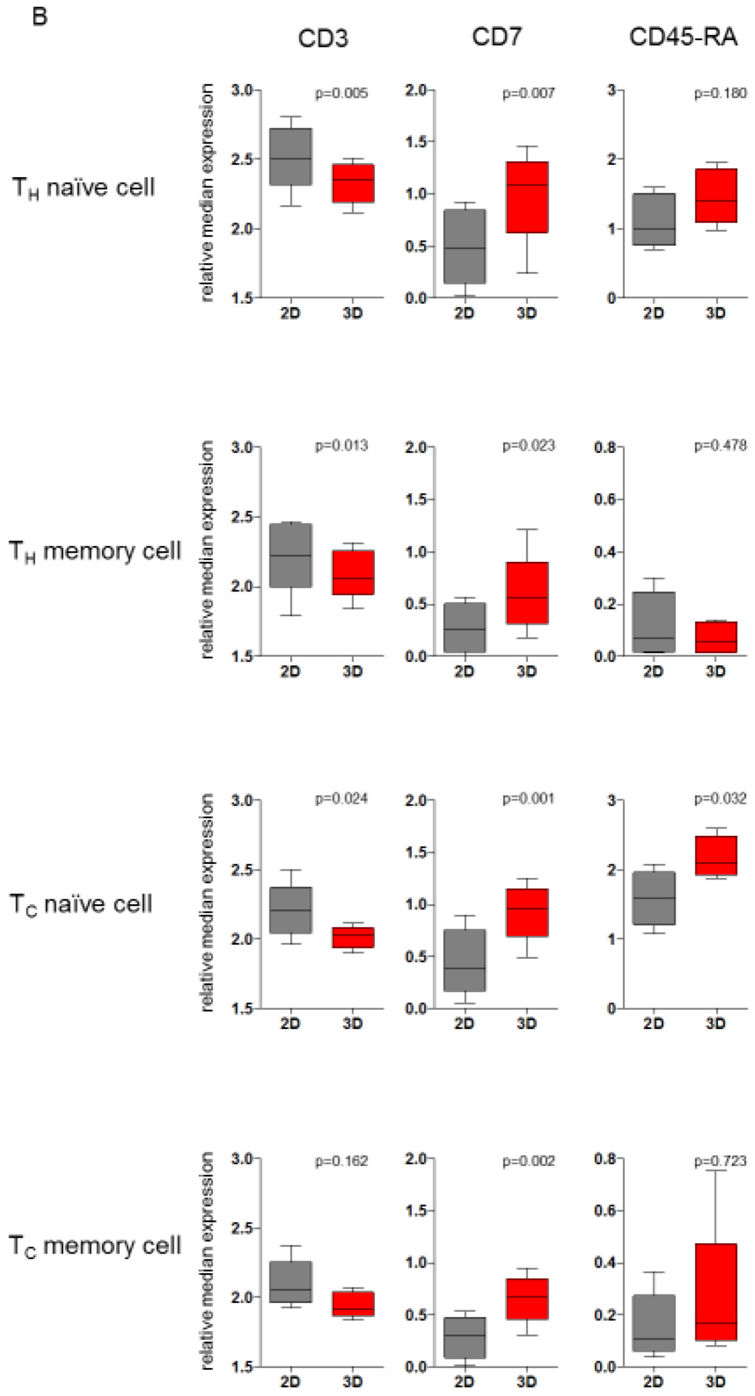

Supplementary Figure S4: T cell subpopulations in 3D vs 2D models by SPADE analysis. B. Relative median expression of T cell specific markers (CD3, CD7 and CD45-RA) in T_H and T_C (naïve and memory) immune subsets in 2D and 3D co-culture models is shown (Holm-Sidak of $p < 0.05$ values).
